# Inefficient Nef-Mediated Downmodulation of CD3 and MHC-I Correlates with Loss of CD4^+^ T Cells in Natural SIV Infection

**DOI:** 10.1371/journal.ppat.1000107

**Published:** 2008-07-18

**Authors:** Michael Schindler, Jan Schmökel, Anke Specht, Hui Li, Jan Münch, Mohammad Khalid, Donald L. Sodora, Beatrice H. Hahn, Guido Silvestri, Frank Kirchhoff

**Affiliations:** 1 Institute of Virology, University of Ulm, Germany; 2 Departments of Medicine and Microbiology, University of Alabama at Birmingham, Birmingham, Alabama, United States of America; 3 University of Texas Southwestern Medical Center, Dallas, Texas, United States of America; 4 Yerkes Regional Primate Research Center, Emory University, Atlanta, Georgia, United States of America; 5 Department of Pathology and Laboratory Medicine, University of Pennsylvania, Philadelphia, Pennsylvania, United States of America; Northwestern University, United States of America

## Abstract

Recent data suggest that Nef-mediated downmodulation of TCR-CD3 may protect SIVsmm-infected sooty mangabeys (SMs) against the loss of CD4^+^ T cells. However, the mechanisms underlying this protective effect remain unclear. To further assess the role of Nef in nonpathogenic SIV infection, we cloned *nef* alleles from 11 SIVsmm-infected SMs with high (>500) and 15 animals with low (<500) CD4^+^ T-cells/µl in bulk into proviral HIV-1 IRES/eGFP constructs and analyzed their effects on the phenotype, activation, and apoptosis of primary T cells. We found that not only efficient Nef-mediated downmodulation of TCR-CD3 but also of MHC-I correlated with preserved CD4^+^ T cell counts, as well as with high numbers of Ki67^+^CD4^+^ and CD8^+^CD28^+^ T cells and reduced CD95 expression by CD4^+^ T cells. Moreover, effective MHC-I downregulation correlated with low proportions of effector and high percentages of naïve and memory CD8^+^ T cells. We found that T cells infected with viruses expressing Nef alleles from the CD4low SM group expressed significantly higher levels of the CD69, interleukin (IL)-2 and programmed death (PD)-1 receptors than those expressing Nefs from the CD4high group. SIVsmm Nef alleles that were less active in downmodulating TCR-CD3 were also less potent in suppressing the activation of virally infected T cells and subsequent cell death. However, only *nef* alleles from a single animal with very low CD4^+^ T cell counts rendered T cells hyper-responsive to activation, similar to those of HIV-1. Our data suggest that Nef may protect the natural hosts of SIV against the loss of CD4^+^ T cells by at least two mechanisms: (i) downmodulation of TCR-CD3 to prevent activation-induced cell death and to suppress the induction of PD-1 that may impair T cell function and survival, and (ii) downmodulation of MHC-I to reduce CTL lysis of virally infected CD4^+^ T cells and/or bystander CD8^+^ T cell activation.

## Author Summary

The accessory Nef protein is commonly considered a “pathogenicity” factor of primate lentiviruses. However, SIVs do not cause disease in their natural hosts, although they all encode *nef* genes and sustain high levels of viremia. To better understand the role of Nef in natural nonpathogenic SIV infection, we compared the function of Nef alleles from two groups of SIVsmm-infected sooty mangabeys: (i) those that maintained normal CD4^+^ T cell counts and (ii) a small subset (10%–15%) of animals that exhibited a considerable loss of CD4^+^ helper T cells. We found that the efficiency of two specific Nef functions, i.e., downmodulation of TCR-CD3 and MHC-I, correlated with preserved CD4^+^ T cell homeostasis, as well as with other immunological features, such as high numbers of proliferating CD4^+^ Ki67^+^ T cells. Moreover, lack of CD3 surface expression was associated with low levels of apoptosis and PD-1 expression by virally infected T cells. Thus, the ability of Nef to remove TCR-CD3 and MHC-I from the cell surface may help the natural hosts of SIV to maintain normal CD4^+^ T cell counts despite high levels of viral replication by preventing activation-induced cell death and CTL lysis of infected T cells and/or CD8^+^ T cell activation.

## Introduction

Although all primate lentiviruses are referred to as human and simian “immunodeficiency” viruses (HIV and SIV, respectively), SIVs do generally not cause disease in their natural monkey hosts [1],[2]. This is in contrast to HIV-1-infected humans and SIVmac-infected macaques, who develop immunodeficiency and AIDS in the absence of antiretroviral therapy [3]. The exact mechanisms underlying nonpathogenic SIV infection are still unclear. Viral loads in naturally infected sooty mangabeys and African green monkeys are just as high as in HIV-1-infected humans and SIVmac-infected macaques [4]–[6]. Furthermore, SIVsmm and SIVagm replicate efficiently in lymphoid tissues and cause massive destruction of CD4^+^ memory T cells in the gut during acute infection [7],[8]. However, a consistent difference between pathogenic and nonpathogenic primate lentiviral infections is that high levels of chronic immune activation associated with accelerated T cell turnover and apoptosis are observed in HIV-1-infected humans and SIVmac-infected macaques but not in naturally SIV-infected primates [1],[2]. Increased immune activation is also a key predictor of progression to AIDS in HIV-1-infected humans [9],[10]. It is thus believed that infection-associated chronic immune activation and T cell apoptosis drive the progressive destruction of the human immune system.

One viral factor that modulates viral fitness, persistence and pathogenicity, is the viral Nef protein. Initially, Nef appeared to represent a potent “virulence” factor because disrupted *nef* genes were associated with very low viral loads and an attenuated clinical course both in HIV-1 and SIVmac infections [11]–[13]. Subsequently, a surprising number of different Nef functions have been described, including modulation of cell surface expression of CD4, CD28, class I MHC and the invariant chain associated with immature class II MHC molecules, as well as enhancement of viral infectivity and replication [14]–[21]. In pathogenic HIV-1 and SIVmac infections, the combination of these Nef functions promotes viral persistance and accelerates disease progression by facilitating viral immune evasion and by enhancing viral spread [22]–[27]. However, high viral loads are not associated with the development of disease in natural SIV infection [1],[2]. Thus, Nef does not act as a pathogenesis factor in natural SIV infections. This may be due to the fact that most primate lentiviral Nef proteins efficiently downmodulate CD3, a key component of the T cell receptor (TCR), from the surface of virally infected T cells, while Nef proteins of HIV-1 and its immediate SIV precursors fail to perform this function [28]. It is controversial whether the HIV-1 Nef enhances or reduces the responsiveness of virally infected T cells to activation [28]–[36]. However, it is clear that *nef* alleles from primate lentiviruses which are capable of downmodulating TCR-CD3 suppress T cell activation and apoptosis substantially more efficiently than those that can not perform this function [28].

Recently, it has been shown that a small subset (10–15%) of naturally infected SMs experience a significant loss of CD4^+^ T cells during the course of their infection [37],[38]. Following up on this observation, we examined the Nef functions of the viruses infecting these animals. Interestingly, we found that efficient Nef-mediated downmodulation of TCR-CD3 was preserved only in those animals with high CD4^+^ T cells, but not in those with low CD4^+^ T cells [28]. This study provided a first indication that Nef may exert a protective role in natural SIV infections; however, it had limitations. First, viruses from only few SMs with low CD4^+^ T cells counts were available for study. Second, Nef functions were only analyzed in Jurkat T cells following transient transfection with vectors coexpressing Nef and eGFP. Finally, only few Nef activities, i.e. downmodulation of CD4, CD3, CD28 and MHC-I, were investigated. In the present study, we examined the effects of *nef* alleles from 11 SMs with CD4^+^ T cell counts >500 cells/µl (CD4high Nef) and 15 SMs with CD4^+^ T cell counts <500 CD4^+^ (CD4low Nef) on the surface expression of various cellular receptors, T cell activation and apoptosis of virally infected cells. Our results show that not only downmodulation of TCR-CD3 but also of MHC-I correlates with the preservation of CD4^+^ T cells in infected managbeys *in vivo*. Thus, primate lentiviral Nef proteins may exert a protective effect in natural SIV infection through two different yet complementary pathways by preventing activation-induced cell death of infected CD4^+^ T cells as well as CD8^+^ T cell activation and CTL lysis.

## Results

### Generation of proviral constructs coexpressing primary SIVsmm Nef alleles and eGFP

To study the function of primary SIVsmm *nef* alleles from SMs with different CD4^+^ T cell counts, we cloned *nef* alleles from 15 animals with low (<500/µl) and 11 animals with high (>500/µl) CD4^+^ T cell counts (CD4low and CD4high Nefs, respectively) into an HIV-1 NL4-3-based IRES-eGFP proviral vector coexpressing Nef and eGFP from bicistronic RNAs [28],[39]. *Nef* alleles were amplified from the plasma of 22 naturally and four experimentally SIVsmm-infected SMs housed at the Yerkes National Primate Research Center (Atlanta, GA) and cloned in bulk into the proviral vector (Figure S1). *Nef* alleles from 19 of these 26 SMs were previously cloned into the bicistronic pCGCG expression plasmid and analyzed in Jurkat T cells by transient transfection [28]. The virological and immunological characteristics of this SM colony, particularly the correlates of CD4^+^ T cell depletion have recently been described [37]. The 15 “CD4low” animals include 12 of 14 previously reported naturally SIVsmm-infected SMs with CD4^+^ cell counts <500/µl [37]. The remaining three CD4low animals were infected by i.v. injection of plasma from a naturally SIVsmm-infected SM [38].

### Molecular characterization of SIVsmm *nef* alleles

**Figure 1:**
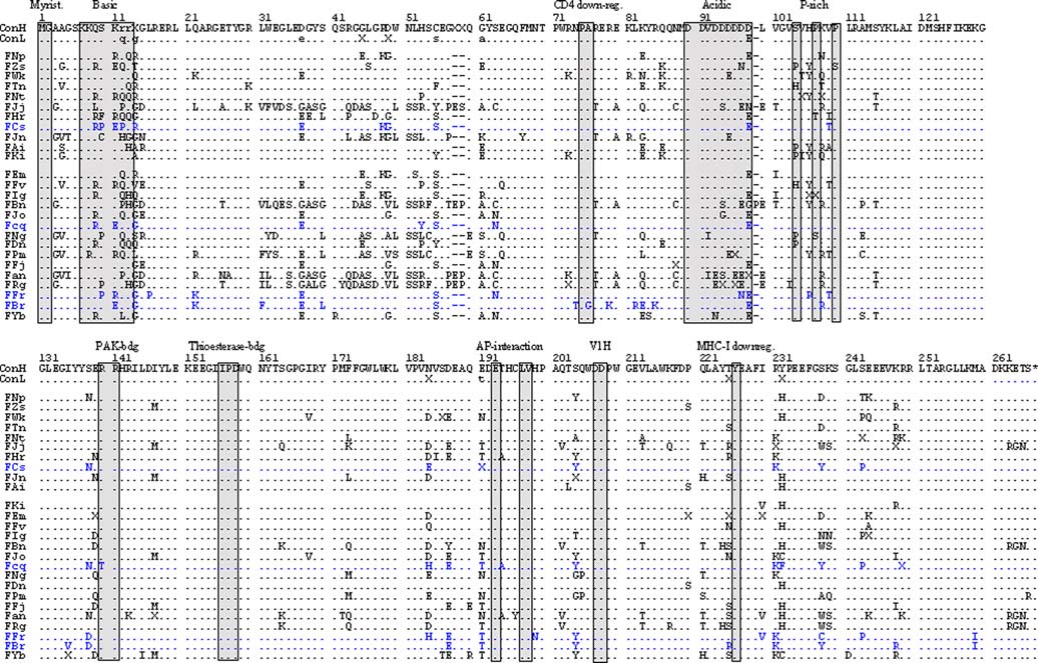
SIVsmm Nef sequences do not contain obvious defects. The SIVsmm consensus Nef amino acid sequences from the CD4high (ConH) and CD4low (ConL) animals are shown in the upper panels. Nef sequences derived from the four experimentally infected SMs are shown in blue. The order of the SIVsmm Nef alleles corresponds to that of the CD4^+^ T cell counts in the respective SMs; i.e. the Nef sequence from animal FNp with the highest numbers shown at the top and from FYb that showest the most severe CD4^+^ T cell depletion at the bottom. Some conserved sequence elements in Nef, including the N-terminal myristoylation signal, N-proximal tyrosines, PA residues critical for CD4 down-modulation by SIVmac Nef, the basic, acidic and proline-rich regions, a diarginine motif, a C-proximal adaptor-protein (AP) interaction site, a diacidic putative V1H binding site and a Y residue involed in MHC-I downregulation are indicated. Dots specify identity with the consensus Nef sequence and dashes gaps introduced to optimize the alignment. PCR-fragments amplified from SM plasma were sequenced directly and “X” indicates nucleotide ambiguity in the corresponding codons.

Phylogenetic analyses of amplified *nef* sequences verified that all proviral plasmid preparations contained *nef* alleles from different animals. Moreover, to ensure appropriate representation of the *nef* alleles present *in vivo* viral stocks were derived from ≥50 independent transformants (data not shown). Alignment of the deduced amino acid sequences showed that several domains and putative protein interaction sites previously described to be relevant for HIV-1 Nef function [40] were conserved in SIVsmm Nefs, including the N-terminal myristoylation signal, the acidic region, a diarginine motif, a C-proximal adaptor-protein interaction site and a diacidic putative V1H binding site (Figure 1). In comparison, the P(xxP)3 motif, which is highly conserved in HIV-1 Nef's and interacts with cellular kinases [40], is not conserved in SIVsmm Nef alleles, which usually contain only two proline residues at this location (Figure 1).

Inspection of the Nef sequences from CD4high and CD4low groups of SMs failed to reveal obvious inactivating mutations, such as large deletions or premature stop codons. However, Nef sequences from the CD4low group were more likely to contain changes in the N-proximal region (residues 31 to 40), the acidic motif and the region downstream of the proline-rich region. Moreover, changes in S63 or E64 were observed in 11 of 15 CD4low but only in 1 of 11 CD4high Nef alleles (Figure 1). Finally, substitutions of S138, E139, E191, Q221 and T225 just upstream of the “diarginine motif”, the “dileucine”-based sorting signal (ExxxLV) [41], and Y226 were substantially more frequent in Nef alleles from the CD4low group of SMs. Y226 corresponds to Y223 in the SIVmac239 Nef and is required for MHC-I downmodulation but not for other Nef activities [24],[42]. Altogether, the sequence comparisons indicated that SIVsmm *nef* genes from SMs with both high and low CD4^+^ T cell counts encoded full-length Nef proteins but frequently differed in amino acid residues flanking known functional motifs.

### Efficient downmodulation of TCR-CD3 and MHC-I correlates with high CD4^+^ T cell counts in SIVsmm-infected SMs

**Figure 2:**
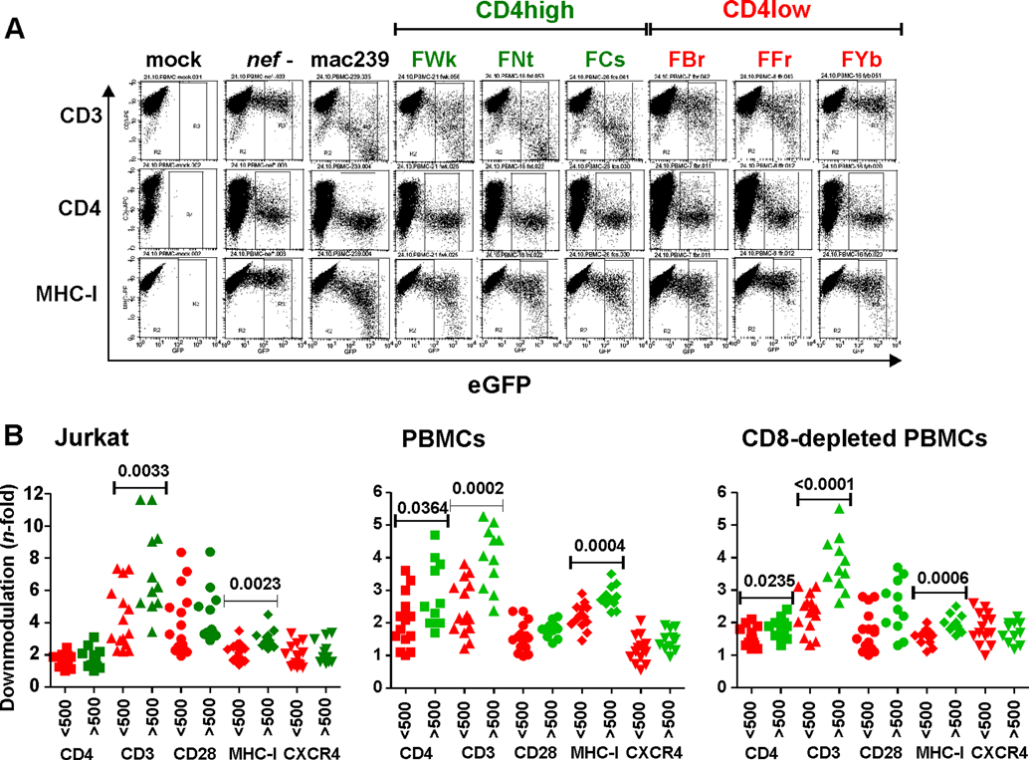
SIVsmm *nef* alleles derived from CD4high SMs are particularly active in downmodulating CD3 and MHC-I. (A) Surface expression of CD3, CD4 and MHC-I on PBMCs infected with HIV-1 Nef/eGFP constructs expressing eGFP alone (*nef*-) or together with indicated *nef* alleles. 239 specifies the SIVmac239 Nef. (B) Quantitative assessment of Nef-mediated downmodulation of the indicated cellular receptors on Jurkat cells, PBMCs and CD8-depleted PBMCs. SIVsmm *nef* genes were grouped by CD4^+^ T cell counts of the animals from which they were amplified and are color coded red and green, respectively. Each symbol represents *n*-fold down-modulation of the indicated receptor molecule by one of the 26 NL4-3 recombinants expressing primary bulk SIVsmm *nef* alleles analyzed. Similar results were obtained in two independent experiments. P-values <0.05 are indicated.

As reported previously [28], the proviral NL4-3 Nef/eGFP constructs are isogenic except for their *nef* coding sequences and have the advantage that Nef expression is mediated by the viral LTR promoter and *via* the normal splice sites. Moreover, pseudotyping with the VSV-G envelope protein bypasses the effect of Nef on virion infectivity [43] and hence allows to transduce primary T cells with comparable efficiency independently of their *nef* coding regions. Finally, since infected cells coexpress Nef and eGFP from single bicistronic RNAs, Nef expression can be correlated with receptor expression levels, activation status and apoptosis [28],[39]. To compare the potency of primary SIVsmm *nef* alleles from SMs with differential CD4^+^ T cell counts in downmodulating CD3, CD4, CD28, MHC-I and CXCR4, we transduced Jurkat T cells with the proviral constructs and analyzed them by flow cytometry (examples shown in Figure S2). Consistent with earlier results [28], we found that CD4high *nef* alleles were significantly more active in TCR-CD3 downmodulation than those from the CD4low animals (7.2±0.8, n = 11 vs 4.2±0.5, n = 15; P = 0.0033; numbers give average n-fold downmodulation ±SD) (Figure 2). However, the present analysis of a larger number of *nef* alleles also revealed significant differences in MHC-I downmodulation between the CD4high and CD4low groups (3.0±0.1, n = 11 vs 2.3±0.1, n = 15; P = 0.0023). Exclusion of *nef* genes from four experimentally infected SMs did not change these results: the differences in CD3 and MHC-I modulation remained significant (P = 0.027 and P = 0.011, respectively). In comparison, the two sets of *nef* alleles did not differ significantly in their abilities to downmodulate CD4, CD28 and CXCR4 from the surface of infected Jurkat cells. Notably, the detectable effect of Nef on CD4 was weak because the proviral constructs express Vpu and Env which also downmodulate this receptor [44],[45]. Since SIVsmm specific Nef antibodies are not available, the contribution of Nef expression levels to the observed functional differences could not be assessed. However, the fact that the majority of *nef* alleles were functionally active at least in some assays implies that they are efficiently expressed.

Next, we examined Nef function in human PBMCs infected with eGFP-expressing reporter viruses (data summarized in Table S1). Importantly, these experiments were performed in both the presence and absence of CD8^+^ T cells (examples shown in Figure 2A and S2) to (i) maintain some of the complexity that exists *in vivo* and (ii) to ensure that transduction of CD8^+^ T cells (that are usually not infected with SIV and HIV) does not impact the results. Flow cytometric analyses of virally infected PBMCs confirmed the correlation between efficient TCR-CD3 (4.0±0.3 vs 2.4±0.2; P = 0.0002) and MHC-I (2.8±0.1 vs 2.2±0.1; P = 0.0004) downmodulation and preserved CD4^+^ T cell counts (Figure 2B, middle). On average, PBMCs infected with reporter viruses containing CD4high *nef* alleles also expressed lower levels of the CD4 molecule, but this difference was less pronounced. In comparison, the effect of all SIVsmm *nef* alleles on CD28 and CXCR4 surface expression on PBMCs was weak and did not differ significantly between the CD4high and CD4low groups. CD8-depleted PBMC cultures yielded identical results (Figure 2B, right). Finally, correlation analyses demonstrated that all *nef* alleles that efficiently downmodulated CD4, TCR-CD3 and MHC-I in Jurkat cells were also highly active in PBMCs (Figure S3).

**Figure 3:**
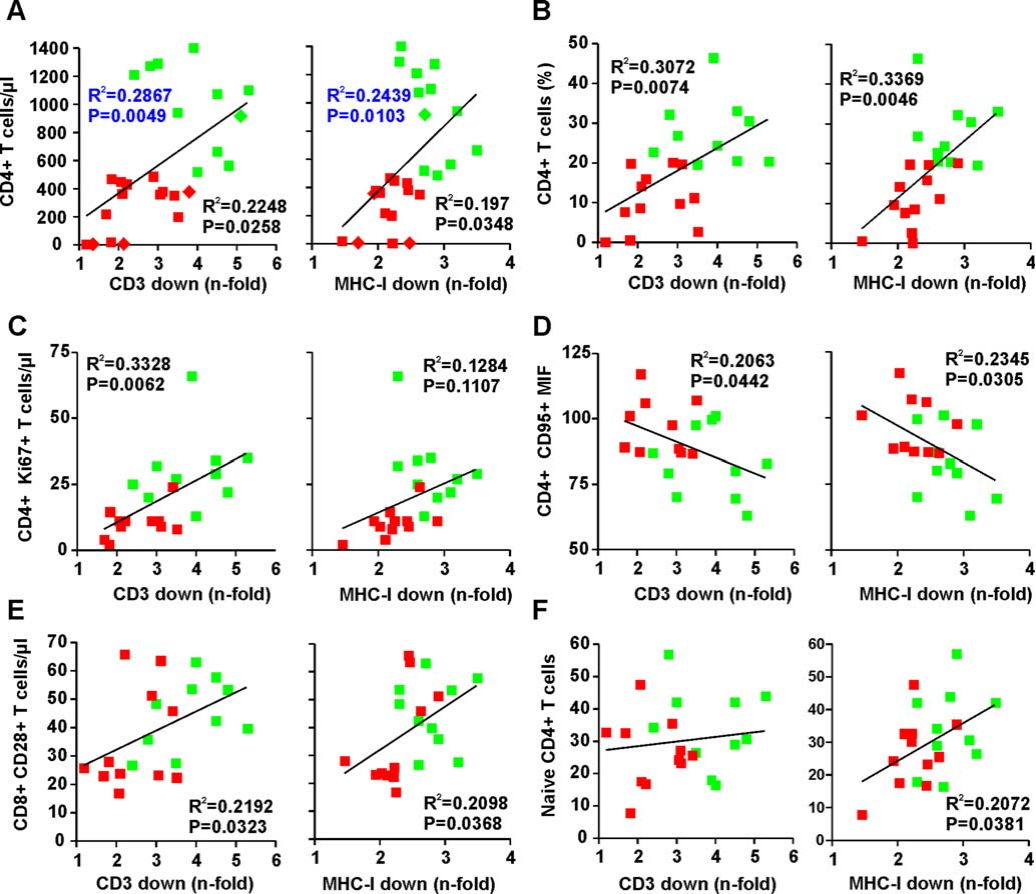
Correlation between Nef-mediated CD3 and MHC-I downmodulation in virally infected PBMCs and immological features in SIVsmm infected SMs. Correlation between (A) the absolute numbers or (B) percentages of CD4^+^ T-cells, (C) number of proliferating CD4^+^Ki67^+^ T cells, (D) CD95 expression levels of CD4^+^ T-cells, (E) number of CD8^+^CD28^+^ T cells and (F) percentage of naïve CD4^+^ T cells in SIVsmm-infected SMs and *n*-fold downmodulation of CD3 and MHC-I. Assays were performed in PBMCs and statistical analysis was performed using the PRISM 4:0 package. The numbers of CD4^+^ T cells obtained from four experimentally infected SMs are indicated by diamond symbold in panel A; all remaining data were derived from naturally SIVsmm-infected animals. P and R^2^ values derived from naturally SIVsmm-infected SMs are shown in black, those including the four experimentally infected animals in blue.

To further evaluate the role of TCR-CD3 and/or MHC-I downmodulation by Nef in the maintenance of stable CD4^+^ T cell numbers, we performed correlation analyses. As shown in Figures 3A and S4A, the efficiency of these Nef functions correlated with the numbers of CD4^+^ T cells in SIVsmm-infected SMs *in vivo*. The correlations between both absolute and relative CD4^+^ T cell counts and the potency of Nef-mediated downmodulation of TCR-CD3 or MHC-I by Nef remained significant when only the 22 naturally SIVsmm-infected animals were included in the analyses (Figures 3A, 3B and S4A, S4B). The strongest correlation between high numbers or percentages of CD4^+^ T cells and Nef function was obtained for the sum of TCR-CD3 and MHC-I downmodulation (Figure S4C, S4D), suggesting that both functions synergize to protect SIVsmm-infected SMs against the loss of CD4^+^ T cells.

### Correlation between Nef function and immunological features of SIVsmm infection

We next examined whether these specific Nef functions correlated with additional virological or immunological parameters determined for this natural SIV infection cohort. As shown in Figures 3C and S5A, we found that the efficiency of Nef-mediated downmodulation of CD3 correlated with higher numbers of proliferating CD4^+^Ki67^+^ cells in SIVsmm-infected SMs. The same trend was observed for modulation of MHC-I by Nef but failed to reach significance (Figures 3C, S5A). In addition, we observed a significant inverse correlation between the efficiency of both TCR-CD3 and MHC-I downmodulation and the levels of CD95 expression by CD4^+^ T cells (Figures 3D, S5B), suggesting that the levels of CD95-mediated apoptosis of CD4^+^ T cells as well as their effect on CD8^+^ bystander T cells [46] may be reduced in SMs infected with SIVsmm Nef variants that effectively downmodulate CD3 and MHC-I. We also found that efficient CD3 and MHC-I downmodulation correlated with higher numbers of CD8^+^CD28^+^ T cells (Figures 3E, S5C). Notably, it has been shown previously that decreases in the absolute numbers of CD8^+^CD28^+^ T cells are predictive for progression to AIDS in HIV-1 infected individuals [47] and associated with declining CD4^+^ T cell counts in SIV-infected SMs [37]. Finally, we observed that the number of naïve CD4^+^ T cells correlates with the efficiency of MHC-I but not TCR-CD3 downmodulation by Nef (Figures 3F, S5D). In contrast, we did not find significant correlations between Nef function and other immological parameters examined, such as the absolute numbers of CD8^+^ T cells and the levels of proliferating (i.e. Ki67^+^) CD8^+^ T cells or of activated (i.e. CD69^+^ or HLA DR^+^), effector (i.e. CD127^−^) and regulatory CD25^+^CD4^+^ T cells.

**Figure 4:**
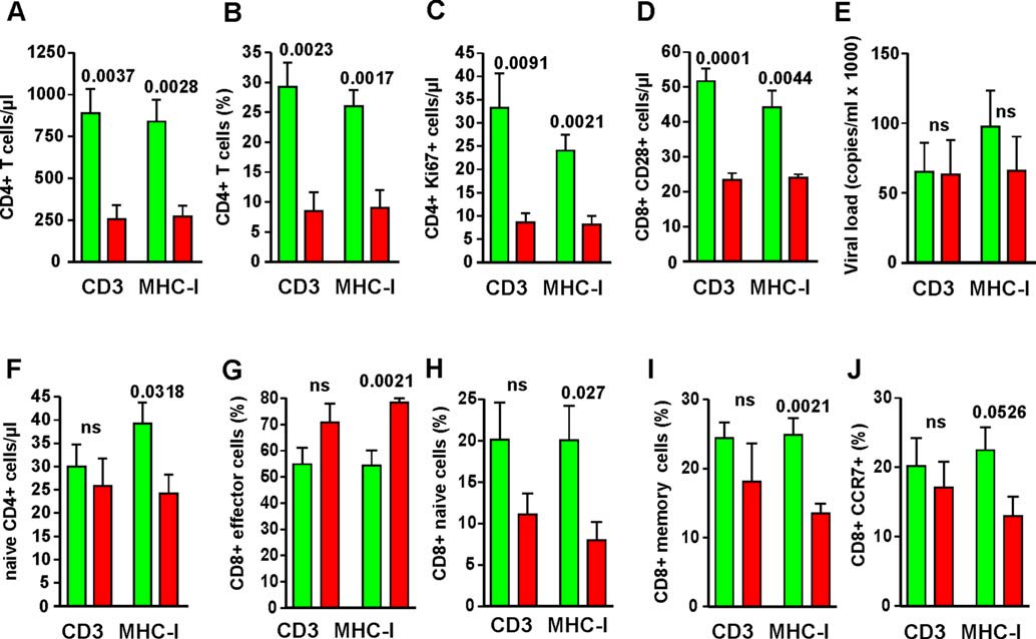
Immunological features in SMs infected with SIVsmm strains differing in their ability to downmodulate TCR-CD3 and MHC-I. Comparison of (A) the absolute numbers or (B) percentages of CD4^+^ T-cells; (C) number of proliferating CD4^+^Ki67^+^ T cells; (D) absolute number of CD8^+^CD28^+^ T cells; (E) viral load; (F) percentages of naïve CD4^+^ and of (G) effector, (H) naïve, (I) memory and (J) CD8^+^ CCR7^+^ T cells cells in SMs naturally infected with SIVsmm expressing *nef* alleles showing the highest (n = 6, green bars) or lowest (n = 6, red bars) activities of the 22 naturally infected animals analyzed. Indicated are average values ±SEM. Numbers above the bars indicate P values; ns, not significant.

To identify further possible consequences of TCR-CD3 and MHC-I downmodulation, we compared the immunological and virological features of SMs naturally infected with SIVsmm expressing *nef* alleles showing the highest (n = 6) or lowest (n = 6) activities in these two functions, i.e. >4.0- vs <2.1-fold and >2.8- vs <2.2-fold downmodulation in PBMCs. While the results of subgroup analyses need to be interpreted with caution they are a meaningful approach to generate and assess new hypotheses [48]. This analysis confirmed that lack of these Nef functions correlates with reduced numbers and/or percentages of CD4^+^ T cells, CD4^+^Ki67^+^ and CD8^+^CD28^+^ T cells in SIVsmm-infected SMs (Figure 4A–D) but not with significant differences in viral loads (Figure 4E). As expected from the results of the correlation analyses (Figure 3F), efficient MHC-I but not TCR-CD3 downmodulation by Nef was associated with a reduced number of naïve CD4^+^ T cells in SIVsmm-infected SMs (Figure 4F). Furthermore, this comparative analysis showed that the percentage of effector CD8^+^ T cells is significantly lower and that of naïve and memory CD8^+^ T cells significantly higher in animals infected with SIVsmm strains that effectively downregulated MHC-I (Figure 4G–I). We also observed a trend for higher percentages of CD8^+^CCR7^+^ T cells (Figure 4J). Taken together, these data demonstrate that effective TCR-CD3 and MHC-I downmodulation by Nef is a strong correlate of preserved CD4^+^ T cells counts in natural SIVsmm infection of SMs and further support that inefficient MHC-I antigen presentation by virally infected cells affects the CD8^+^ T cell response.

### Nef-mediated suppression of T cell activation and apoptosis correlates with preserved CD4^+^ T cell counts in SMs

**Figure 5:**
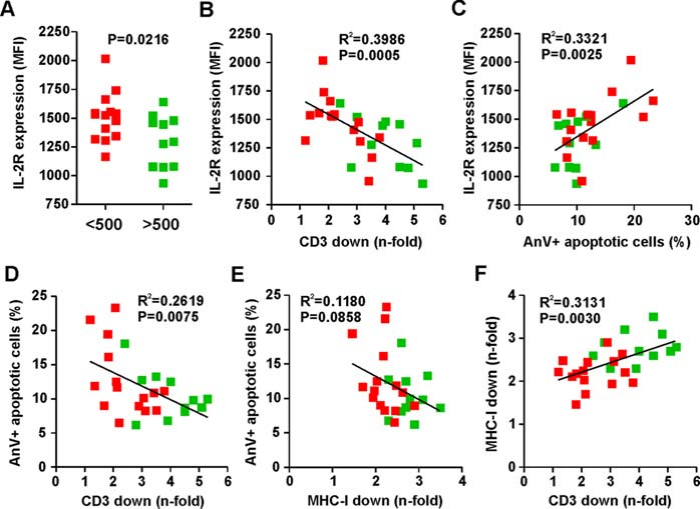
Effect of SIVsmm Nef alleles on IL-2R expression and apoptosis. (A) Levels of IL-2R expression on PBMCs transduced with HIV-1 constructs expressing Nef alleles from SIVsmm-infected SMs with low or high CD4^+^ T-cell counts. The mean fluoresence activities (MFIs) of IL-2R expression were determined at 4 days post-stimulation. (B, C) Correlation between IL-2R expression levels and (B) the efficiency of Nef-mediated CD3 downmodulation or (C) the percentage of apoptotic cells (n = 22). (D, E) Correlation between (D) TCR-CD3 and (E) MHC-I downmodulation and apoptosis. (F) Correlation between TCR-CD3 and MHC-I downmodulation.

We have previously shown that TCR-CD3 downmodulation by Nef blocks the responsiveness of virally infected T cells to activation [28]. To assess whether the relatively subtle functional differences of primary SIVsmm *nef* alleles in TCR-CD3 modulation are also associated with differential responsiveness of infected T cells to stimulation we first determined the surface expression levels of CD69 and of the IL-2 receptor-α chain (IL-2Rα) or CD25, well known markers for early and late T cell activation, in HIV-1-infected PHA-treated regular and CD8-depleted PBMC cultures. These analyses showed that T cells infected with viruses expressing CD4high Nef alleles expressed significantly lower levels of activation markers than those infected with CD4low Nef constructs (Figures 5A, S6A). In agreement with our previous data [28], suppression of CD69 and IL-2R expression correlated with effective downmodulation of TCR-CD3 (Figures 5B, S6B, S6C) and reduced levels of programmed death (Figure 5C). Furthermore, Nef-mediated downmodulation of CD3 (Figure 5D) but not that of MHC-I (Figure 5E), correlated with reduced percentages of apoptotic cells. Although downmodulation of CD3 and MHC-I are mediated by different domains in Nef [24], [26], [49]–[51], both functions correlated with one another (Figure 5F), suggesting that both may be coselected in SIVsmm-infected SMs.

**Figure 6:**
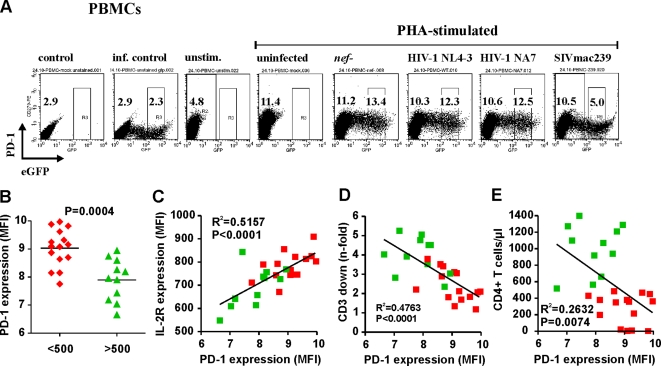
Nef-mediated TCR-CD3 downmodulation correlates with suppression of PD-1 expression. (A) Surface expression of PD-1 on PBMCs that were either not stimulated (unstim.) or stimulated with PHA for 2 days and either not infected or transduced with HIV-1 Nef/eGFP constructs expressing eGFP alone (*nef*-) or together with the NL4-3 or mac239 *nef* alleles. eGFP expression levels used to calculate receptor down-modulation and the MFIs are indicated. Control, unspecific background. (B) Quantitative assessment of PD-1 expression on PBMCs infected with HIV-1 constructs expressing SIVsmm *nef* genes from the CD4low and CD4high groups of SMs. Each symbol represents the PD-1 expression level on PBMCs infected by one of the 26 NL4-3 recombinants expressing primary bulk SIVsmm *nef* alleles analyzed. Similar results were obtained in two independent experiments. (C, D) Correlation between (C) the levels of IL-2R and PD-1 expression on HIV-1 infected PBMCs and (D) PD-1 surface levels and the efficiency of CD3 downmodulation by the corresponding Nef alleles. (E) Correlation between PD-1 expression by virally infected PBMCs in vitro and the CD4+ T cell counts in SIVsmm-infected SMs.

Recent findings suggest that high levels of programmed death (PD)-1 receptor expression contribute to the exhaustion of HIV-1-specific CD8^+^ T cells because blocking PD-1-PD-1 ligand interaction enhanced their ability to proliferate and to produce cytokines in HIV-1 infected individuals [52],[53]. PD-1 is also expressed on activated CD4^+^ T cells [54],[55] and its blockade partly restored CD4 proliferative responses in AIDS patients [52]. It has been shown that PD-1 is inducibly expressed on activated T cells [56]. Since CD3 downmodulation prevents T cell activation, we examined whether this Nef function affects the induction of PD-1 expression by virally infected T cells. We found that PHA stimulation induced a 2.5- to 4-fold increase in PD-1 expression levels (example shown in Figure 6A). Infection with HIV-1 constructs expressing no Nef or Nef alleles that do not downmodulate TCR-CD3 resulted in slightly increased levels of PD-1 expression. In strict contrast, those that downregulated TCR-CD3 generally suppressed the induction of this receptor (Figure 6A). Further analyses demonstrated that Nef alleles from the CD4high group of SMs suppressed the induction of PD-1 expression significantly more efficiently than those derived from the CD4low group of animals (Figure 6B). Since both represent T cell activation markers, we observed a highly significant correlation between the levels of IL-2R and PD-1 expression on HIV-1 infected PBMCs (Figure 6C). Suppression of the induction of PD-1 expression correlated with the efficiency of TCR-CD3 downmodulation (Figure 6D). The correlation between the ability of Nef to downmodulate CD3 and to suppress the induction of CD69, PD-1 and IL-2R was confirmed using CD8-depleted PBMCs (Figure S6). Thus, the ability of Nef to modulate TCR-CD3 may impact the functionality, proliferation and survival of T cells by affecting the expression levels of PD-1 on virally infected CD4^+^ cells.

### *Nef* alleles from an SM with extremely low CD4^+^ T cell counts render T cells hyper-responsive to activation

**Figure 7:**
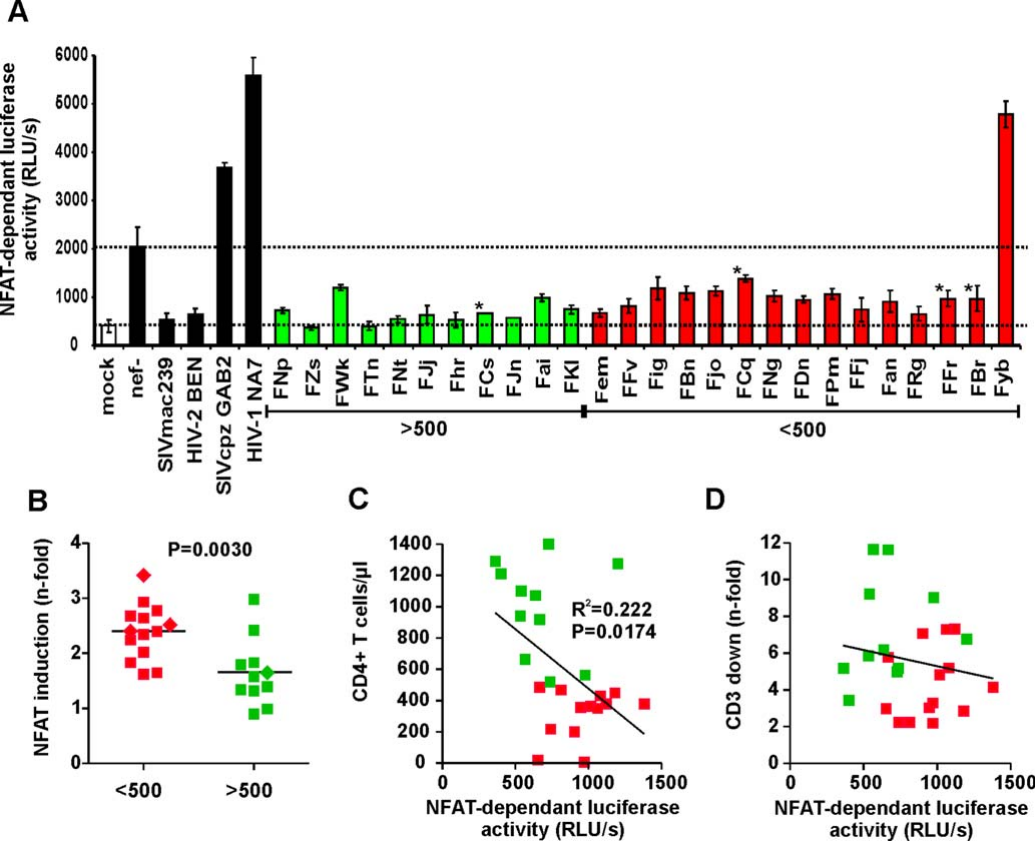
Impact of SIVsmm Nef alleles on NFAT induction. (A) Analysis of Jurkat cells stably transfected with an NFAT-dependent reporter gene [31] following transduction with the indicated HIV-1 Nef/eGFP constructs and subsequent stimulation with PHA. Levels of NFAT-dependent luciferase reporter activity are the average (±SD) of triple infections. Similar results were obtained in two independent experiments. Mock specifies uninfected control cells. The levels of luciferase activity in mock and *nef*-defective HIV-1 infection are indicated by broken lines. (B) N-fold induction of NFAT in T cell cultures infected with HIV-1 variants expressing Nef alleles from SIVsmm-infected SMs with low (n = 14) or high (n = 11) CD4^+^ T-cell counts compared to the mock control. Squares indicate Nef alleles derived from naturally infected SMs and diamonds those from experimentally infected animals. Animal FYb (see panel A) was excluded from the analyses shown in panels B–D. (C, D) Correlation between (C) the CD4^+^ T cell counts in SIVsmm infected SMs and (D) the efficiency of Nef-mediated CD3 downmodulation and PHA-induced levels of NFAT-dependent luciferase activities (*n* = 25).

The nuclear factor of activated T cells (NFAT) regulates transcription of IL-2 gene expression, a hallmark of T cell activation. It has been established that NFAT activation is suppressed by Nef alleles that downmodulate TCR-CD3, e.g. SIVsmm and HIV-2 Nefs, and enhanced by Nef alleles that do not modulate this receptor, such as those of HIV-1 [28],[31]. To assess whether *nef* alleles from the CD4high and CD4low groups of SIVsmm-infected SMs differentially affect NFAT induction in virally infected cells, we transduced Jurkat T cells stably transfected with the luciferase gene under the control of an NFAT-dependent promoter [31], with the proviral HIV-1 eGFP/Nef constructs and examined their responsiveness to activation. T cells infected with *nef* defective HIV-1 showed about 5-fold enhanced levels of NFAT activity after PHA stimulation compared to mock infected cells (Figure 7A). This increase was usually further enhanced by 2- to 3-fold by expression of HIV-1 and SIVcpz Nef alleles but blocked by the control SIVmac and HIV-2 Nefs (examples shown in Figure 7A). SIVsmm Nef alleles from 25 of the 26 infected SMs also suppressed NFAT induction, albeit with differential efficiency. Interestingly, *nef* alleles from animal FYb, that showed the most severe CD4^+^ T cell depletion (3/µl) of the entire colony of 110 naturally SIVsmm-infected SMs [37], rendered the Jurkat T cells hyper-responsive to activation - just like those of HIV-1 (Figure 7A). This unusual property of bulk FYb Nef alleles, which were generally poorly active in other functions including downmodulation of CD3 and MHC-I, was confirmed using 18 HIV-1 IRES/eGFP constructs expressing individual FYb derived Nef proteins (data not shown).

Compared to FYb, the NFAT-dependent luciferase activities were 4- to 13-fold lower in Jurkat cells infected with viral constructs expressing Nef alleles derived from the remaining 25 SMs. However, CD4low Nef alleles were less potent in suppressing NFAT induction than those from the CD4high group (Figure 7B). The levels of NFAT activation in HIV-1-infected T cells inversely correlated with the CD4^+^ T cell counts of the respective SIVsmm-infected SMs *in vivo*, although this correlation was not very stringent (Figure 7C). In comparison, the ability of these 25 *nef* alleles to suppress NFAT induction upon PHA stimulation did not correlate with their potency in downmodulating TCR-CD3 (Figure 7D). This is most likely due to the fact, that the functional differences in modulation of TCR-CD3 between these SIVsmm *nef* alleles are much more subtle than those between Group 1 and 2 *nef* alleles [28], and that other Nef functions, such as downmodulation of CD4, CD28 and CXCR4, also affect the responsiveness of virally infected T cells to activation. Nevertheless, these results further support that high responsiveness of infected T cells to activation accelerates the loss of CD4^+^ T cells in naturally SIV-infected SMs.

### Enhancement of virion infectivity by primary SIVsmm *nef* alleles

**Figure 8:**
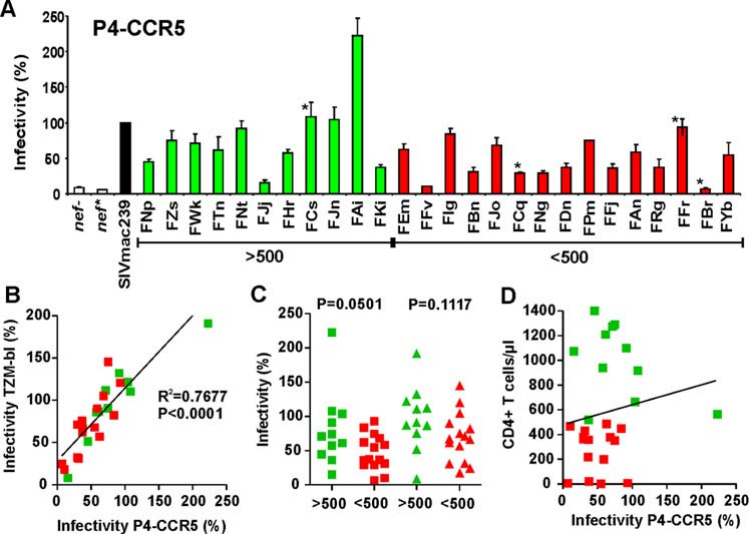
Enhancement of viral infectivity by primary SIVsmm Nef alleles. (A) P4-CCR5 indicator cells were infected with HIV-1 NL4-3 IRES-eGFP constructs containing the indicated SIVsmm *nef* genes or defective *nef* alleles. Infections were performed in triplicate with two different virus stocks containing 1 ng p24 antigen. Shown are average values of the 6 measurements ±SD compared to the infectivity of the virus expressing the SIVmac239 Nef (100%). *Nef* alleles derived from experimentally infected animals are indicated by stars. (B) Correlation between the infectivity of HIV-1 NL4-3 IRES-eGFP constructs expressing primary SIVsmm Nef alleles (n = 26) in P4-CCR5 and TZM-bl cells. (C) Comparsion of virion infectivity enhancement by SIVsmm Nef alleles from animals with high (green) or low (red) numbers of CD4^+^ T cells. (D) Correlation between the number of CD4^+^ T cells in SIVmm infected SMs and the infectivity of the respective HIV-1 IRES-eGFP *nef* recombinants (n = 26).

In addition to facilitating viral immune evasion, Nef has been reported to also enhance virion infectivity [14],[15],[17],[18]. To examine whether CD4^+^ T cell loss in SIVsmm-infected SMs is associated with differences in this Nef function, we exposed the HeLa-CD4/LTR-lacZ indicator cell line, P4-CCR5, which expresses CD4 and both major entry cofactors CXCR4 and CCR5 [57],[58], to 293T cell-derived virus stocks and determined the β-galactosidase activities after two days. Most bulk SIVsmm *nef* alleles enhanced virion infectivity, albeit with varying efficiencies (Figure 8A). Analysis of the same reporter viruses using the TZM-bl indicator cell line, previously designated JC53-bl [59],[60], yielded the same results (Figure 8B). On average, *nef* genes from SIVsmm-infected SMs with high CD4^+^ T cell counts were slightly more active in enhancing virion infectivity than those from animals with low numbers of CD4^+^ T cells (Figure 8C). However, this difference was mainly due to the unusually high activity of *nef* alleles from a single animal (FAi) and not significant. Moreover, the potency of Nef in enhancing virion infectivity did not correlate with the CD4^+^ T cell counts in SIVsmm-infected SMs (Figure 8D).

### Impact of specific amino acid substitutions on SIVsmm Nef function

To analyze the effect of sequence variations between the CD4high and CD4low groups of SMs on Nef function we synthesized the consensus *nef* sequences derived from the six animals with the highest (SMhi) and the six SMs with the lowest (SMlow) numbers of CD4^+^ T cells, which differed in a total of 20 amino acid residues (Figure S7A). We found that the SMhi Nef allele was moderately more active in downmodulating CD3 and MHC-I and in suppressing T cell activation and programmed death than the SMlow consensus Nef (Table S2). Thus, the SMhi and SMlow Nef alleles exhibited functional differences reflecting those observed for the primary *nef* alleles derived from SMs with high and low CD4^+^ T cell counts. However, both consensus Nefs were more active than most primary Nef alleles. This phenotype is similar to that of consensus Nef alleles from HIV-1-infected individuals with progressive and nonprogressive infection, which also displayed unusually high activity and only subtle functional differences [23]. The reason for this phenotype is most likely that these consensus Nefs contain optimal amino acid residues at most positions, although the possibility that they may be expressed at particularly high levels cannot be exluded.

**Figure 9:**
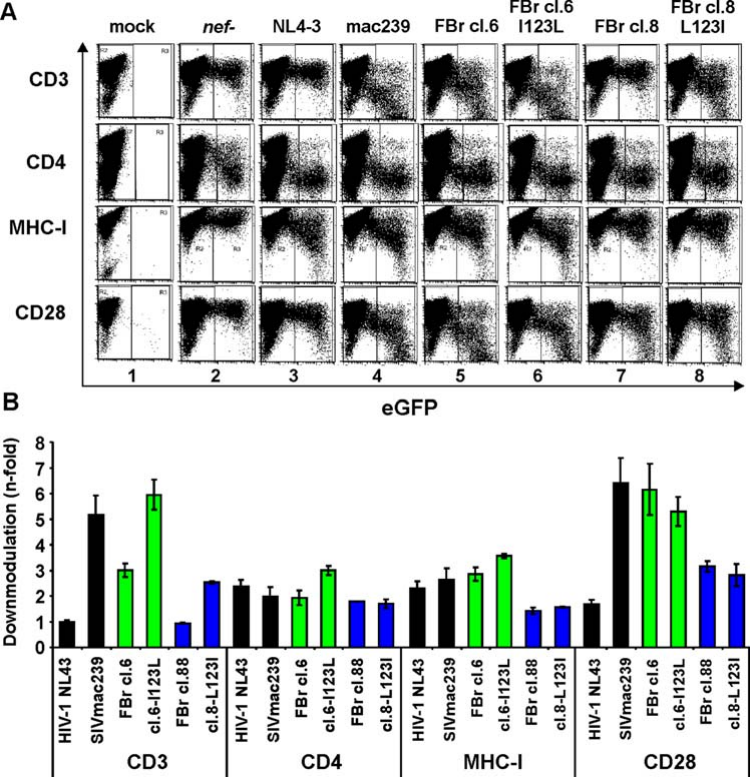
Characterization of an SIVsmm *nef* allele selectively impaired in CD3 downmodulation. (A) Surface expression of CD3, CD4 and MHC-I on PBMCs infected with HIV-1 Nef/eGFP constructs expressing eGFP alone (*nef*-) or together with indicated *nef* alleles. (B) Quantitative assessment of Nef-mediated down-modulation of the indicated cellular receptors on PBMCs. Shown are averages ±SD derived from two independent experiments.

To further assess which amino acid variations modulate the ability of Nef to downregulate CD3 and MHC-I we analyzed about 50 individual primary SIVsmm *nef* alleles. The results confirmed the data obtained from the analyses of Nef function in bulk. For example, the FCs clone 1 Nef allele derived from a CD4high SM efficiently downmodulated CD3 and suppressed T cell activation, whereas the FYb clone 17 Nef from a CD4low animal was poorly active (Table S2). To map residues underlying these functional differences, we replaced eight amino acid residues in the FCs clone 1 Nef with the corresponding residues in FYb and introduced the reverse changes in the FYb clone 17 Nef (Figure S7B). Our data showed that these changes impaired the activity of the FCs clone 1 Nef in receptor downmodulation but rendered it active in causing hyper-responsiveness of T cells to activation–similarly to those of FYb. In contrast, the reverse changes partially restored the ability of the FYb clone 17 Nef to downmodulate CD3 and to suppress T cell activation and programmed death (Table S2). Interestingly, one of 15 *nef* alleles (clone 8) from another animal (FBr) was impaired in CD3 but not in CD4 or CD28 downmodulation (Figure 9A). Since it differed in only eight amino acid residues from active FBr Nef alleles (Figure S7C; Table S2), we introduced all eight changes (five individually and the RKT to HRV substitution in the “P-rich” region in combination) into the FBr clone 8 Nef. Unexpectedly, only a homologous L123I change specifically enhanced its activity in CD3 downmodulation, while the reverse I123L substitution in the active FBr clone 6 Nef increased its ability to modulate CD3 even further (Figure 9A; Table S2). Thus, although the determinants of CD3 downmodulation by SIVsmm Nef are obviously complex and dependent on the specific Nef backbone, residue I123 seems to be specifically involved in Nef-mediated modulation of CD3 but not of other surface receptors. Further mutational analyses of SIVsmm Nef alleles differing in specific functions due to a limited number of amino acid substitutions should allow to pinpoint the critical residues.

## Discussion

In the present study we performed a detailed functional analysis of Nef proteins from naturally infected SM with different infection outcomes. Our results show that inefficient downmodulation of TCR-CD3 by SIVsmm Nef alleles correlated with the loss of CD4^+^ T cells. In other words, a more “HIV-1-like” Nef phenotype of SIVsmm, i.e. the inability to efficiently downregulate TCR-CD3, was associated with a course of SIVsmm infection that was more reminiscent of pathogenic HIV-1 infection. At first view it may seem paradoxical that downmodulation of TCR-CD3 - a molecular complex essential for the function of T cells–may protect against the development of immunodeficiency. However, at least during chronic infection only a small fraction of CD4^+^ T cells is productively infected. Thus, it is conceivable that their functionality is not critical for the overall immune competence of the infected host. The rate at which these virally infected T cells die and must be replaced, however, must have a significant impact on the host's regenerative capacity. Furthermore, CD4^+^ helper T cells orchestrate the immune response and are typically able to exert significant effects on the immune system by relatively small numbers. Thus, virally infected activated CD4^+^ T cells likely contribute to high levels of immune activation by sequestering cytokines that induce the migration and inflammatory response of uninfected bystander cells. It is thus possible that a Nef-mediated suppression of CD4^+^ T cell activation and cell death contributes to a general reduction of infection-associated immune activation. Notably, our findings likely underestimate the real impact of Nef's ability to downmodulate TCR-CD3 on the rates of CD4^+^ T cell depletion because the functional differences between *nef* alleles from the CD4low and CD4high groups of SIVsmm-infected SMs are substantially more subtle than those between HIV-1 and the great majority of SIVs that are usually highly effective in removing TCR-CD3 from the cell surface [28].

In our previous study [28], we observed a nonsignificant trend for more efficient MHC-I downmodulation by CD4high Nefs. The present analysis of a larger number of SIVsmm infections now reveals a significant correlation between efficient MHC-I downmodulation and stable high CD4^+^ T cell counts. A protective role of this Nef function is consistent with the observation that HIV-1 *nef* alleles from some long-term nonprogressors remain competent in downregulating MHC-I but are otherwise defective [61],[62]. We also found that effective Nef-mediated MHC-I downmodulation is associated with low percentages of effector but high proportions of naïve and memory CD8^+^ T cells (Figure 4G–I). This is in agreement with published data showing that weak MHC-antigen stimulation of CD8^+^ naïve precursors favors the generation of memory rather than effector CD8^+^ T-cells [63] and that SIVmac mutants selectively impaired in MHC-I downmodulation cause stronger CD8^+^ T-cell responses [26]. Finally, efficient downmodulation of both CD3 and MHC-I by Nef correlated with higher numbers of CD8^+^CD28^+^ T cells. While further studies are required to understand the functional significance of these associations, it is tempting to speculate that efficient MHC-I downregulation by Nef may contribute to the maintenance of high CD4^+^ T cells in naturally infected primates by two different mechanisms, i.e. by reducing CTL lysis of virally infected CD4^+^ T cells and by diminishing the levels of CD8^+^ bystander T cell activation.

Accumulating evidence suggests that primate lentiviral Nef proteins generally promote viral persistance in the infected host by facilitating immune evasion (i.e. by modulation of CD4, MHC-I and Ii surface expression) and by enhancing viral infectivity and replication [28],[64],[65]. Thus, Nef is most likely critical for the development and maintenance of high viral loads in natural as well as in recent and nonnatural SIV and HIV infections. However, Nef proteins from most SIVs and from HIV-2 also appear to exert a protective effect that is linked to their ability to downmodulate CD3 from the cell surface. As shown in the present study, lack of this Nef function is correlates with a decline in CD4^+^ T cells in primates that usually do not loose their CD4^+^ T cells as a consequence of SIV infection. Whether the reduced ability of Nef to downmodulate CD3 and MHC-I is the cause or consequence of CD4^+^ T cell decline remains to be investigated. However, there is no evidence that the quality or the potency of the antiviral immune response differs in SMs with high or low CD4^+^ T cell counts [37], which argues against the possibility that a more effective immunity drives the observed functional differences in Nef. Our new data are thus in agreement with our previous hypothesis that the evolutionary loss of Nef-mediated downmodulation of TCR-CD3 contributes to the escalation of immune activation and the progression to AIDS in HIV-1-infected individuals [28]. The fact that SIVmac is pathogenic in macaques (although its Nef downmodulates CD3) does not argue against this hypothesis. Firstly, this virus does not cause disease or high levels of immune activation when reintroduced into its original host, the sooty mangabey [6],[66],[67]. Thus, the pathogenic phenotype in macaques seems to be a consequence of an unusually high susceptibility of this nonnatural host species to SIV-induced disease rather than an inherent viral phenotype. Secondly, even in the SIV/macaque model increased T cell activation by Nef further accelerates disease progression [22]. Notably, *nef*-deleted attenuated HIV-1 infection is ultimately pathogenic in humans unless replication is completely suppressed [68]–[70] suggesting that Nef may accelerate disease progression in nonnatural HIV/SIV hosts mainly because it increases the viral loads by several orders of magnitude and not because it enhances virulence directly. These data suggest that in addition to maintaining high viral loads most primate lentiviral Nefs also reduce immune activation and T cell apoptosis and thus the damaging effects associated with these high viral loads by removing TCR-CD3 from the cell surface.

We have previously shown that TCR-CD3 downmodulation by Nef inhibits induction of IL-2R expression, NF-AT activation and activation-induced cell death [28]. Here, we demonstrate that the efficiency of this Nef function also correlates with the suppression of the induction of PD-1 expression on virally infected T cells. The role of this receptor in the pathogenesis of AIDS has received a lot of attention because it has been shown that high levels of PD-1 expression correlate with declining CD4^+^ T cell counts and disease progression and are associated with reversible dysfunction of CD4^+^ and CD8^+^ T cells [52],[53]. We thus anticipated that downmodulation of CD3-TCR would also inhibit the induction of PD-1 expression because this receptor is expressed predominantly on activated cells [54],[55]. This was indeed observed (Figure 6). Furthermore, mutations in Nef disrupting CD3 downmodulation also impaired its ability to suppress PD-1 induction. Conversely, a truncated form of the SIVmac239 Nef (tNef) that is capable of downmodulating CD3 but inactive in modulating CD4, MHC-I and CD28 and enhancing virus infectivity [50], efficiently inhibits PD-1 induction (data not shown). These data suggest that Nef-mediated downmodulation of CD3 may be required and sufficient for the suppression of PD-1 induction, although the possibility that these Nef mutants differ in additional functions cannot be excluded. It has been reported that PD-1 inhibits the proliferation of T cells and sensitizes them for apoptosis [46],[71],[72]. Thus, high levels of PD-1 expression should lead to increased programmed death of infected T cells and may hence accelerate their depletion. In agreement with this possibility we found that Nef alleles from SMs experiencing a significant loss of CD4^+^ T cells suppressed the induction of PD-1 less efficiently than those derived from animals with stable CD4^+^ T cell counts. Notably, upregulation of PD-1 on virally infected CD4^+^ T cells may also affect the function of bystander T and B cells via interactions with its ligands PD-L1 and PD-L2 [54]–[56]. Thus, further studies are required to clarify how differential levels of PD-1 expression on T cells infected with primate lentiviruses that do or do not downmodulate TCR-CD3 affect the immunological and clinical outcome of infection.

In HIV-1-infected humans, high levels of proliferating CD4^+^Ki67^+^ T cells correlate with disease progression [73],[74]. However, no correlation between the level of CD4^+^Ki67^+^ T cells and signs of disease progression or CD4^+^ T cell depletion was observed in SIV-infected SMs [37]. Unexpectedly, we found significantly higher numbers of proliferating CD4^+^Ki67^+^ T cells in animals infected with SIVsmm Nef variants that maintained high levels of CD4^+^ T cells despite the fact that Ki67 is usually seen as an activation marker. Since downmodulation of TCR-CD3 and MHC-I also correlates with reduced expression levels of CD95 on CD4^+^ T cells (Figures 3D, S5B), the higher levels of CD4^+^Ki67^+^ T cells may simply reflect the prolonged survival of proliferating CD4^+^ T cells due to reduced levels of apoptosis. As discussed above, increased PD-1 expression is associated with reduced proliferative capacity [47],[52],[53]. Thus, it is tempting to speculate that Nef-mediated suppression of PD-1 may also contribute to the high levels of CD4^+^Ki67^+^ T cells. It has been proposed that enhanced levels of apoptosis of bystander CD8^+^ T cells are important for the development of AIDS in HIV-1-infected humans and SIV infected monkeys [46],[75]. Thus, it will be of interest to investigate whether reduced CD95 expression on CD4^+^ T cells in SMs infected with SIVsmm variants that effectively downmodulate TCR-CD3 and MHC-I is associated with reduced apoptosis of bystander T cells expressing the CD95 ligand.

A striking finding of the present study was that one naturally infected SM with very low CD4^+^ T cell numbers was the only one of 26 animals investigated which was infected with an SIVsmm strain whose Nef caused hyper-responsiveness of virally infected T cells to activation, a phenotype previously established for HIV-1 Nef alleles [31]. It is conceivable that direct enhancement of the responsiveness of virally infected T cell to activation by Nef may enhance activation-induced cell death and hence accelerate the loss of CD4^+^ T cells more dramatically than just inefficient inhibition of these processes. It is noteworthy, however, that this animal (FYb) did not show unusually high levels of immune activation and was already of old age [37]. Thus, further studies are required to clarify the role of Nef's ability to alter the responsiveness of infected T cells to activation in the course of primate lentiviral infection; i.e. it would be of high interest to assess whether infection of SMs with SIVsmm expressing the FYb *nef* allele experience a rapid loss of CD4^+^ T cells. Notably, the severe and persistent loss of CD4^+^ T cells in two of the four experimentally infected SMs examined was not only associated with inefficient downmodulation of TCR-CD3 but also with the emergence of SIVsmm variants capable to utilize CXCR4 and CCR8 in addition to CCR5 [38]. We have initiated studies to determine the temporal pattern of changes in Nef function and coreceptor usage and to clarify how the changes in these viral properties correlate with the loss of CD4^+^ T cells. Altogether, these data suggest that viral properties that are uncommon for SIVs but not for HIV-1, i.e. an expanded coreceptor tropism and inefficient suppression of T cell activation, are associated with CD4^+^ T cell depletion even in naturally SIV-infected. Interestingly, none of the SIVsmm-infected SMs with persistent dramatic CD4+ T cell depletion developed AIDS [37],[38]. Thus, further studies to elucidate which viral or host factors prevent disease progression in SIVsmm-infected SMs, even following dramatic CD4^+^ T cell depletion, are highly warranted.

In summary, our data suggest that - in addition to other host and viral properties [1],[2],[76] - differences in specific Nef functions may modulate the immunological outcome of primate lentiviral infections. In particular, Nef-mediated downmodulation of TCR-CD3 and MHC-I seem to protect naturally infected SMs against loss of CD4^+^ T cells. Both of these functions presumably delay the apoptotic death of virally infected T cells, either by suppressing their activation or by preventing CTL lysis. However, these Nef functions may also affect the activation and programmed death of uninfected bystander T cells. The latter is supported by our new finding that efficient Nef-mediated MHC-I downmodulation is associated with low numbers of effector and high numbers of memory CD8^+^ T cells. At this point, our data are no definitive proof that there is a direct causal relationship between Nef-mediated downmodulation of CD3 and MHC-I and preserved CD4^+^ T cell homeostasis. Instead this will require the testing of selective SIV Nef mutants *in vivo* in appropriate SIV/monkey models, such as SIV-infected SMs or AGMs. Such experiments should also help to elucidate the role of T cell activation and/or CTL lysis in CD4^+^ T cell depletion and to assess whether preventing hyperactivation of the immune response would improve current treatment strategies.

## Methods

### Animals

Blood samples were obtained from 22 naturally and four experimentally SIVsmm-infected SMs, all housed at the Yerkes National Primate Research Center of Emory University and maintained in accordance with NIH guidelines. These studies were approved by the Emory University Institutional Animal Care and Use Committee (IACUC). The virological and immunological characteristics of these animals have recently been described [37],[38]. Plasma viral RNA was extracted using the QIAamp Viral RNA Kit (Qiagen) and *nef* genes were amplified by RT-PCR using primers SM-Nef-F1 (5′-GACAGATAGAATATATTCATTTCC-3′) and SM-Nef-R1 (5′-TCTGCCAGCCTCTCCGCAGAG-3′). Sequence analysis of two clones per amplicon (two per animal) confirmed the integrity of the cloned *nef* alleles.

### Proviral constructs

Generation of HIV-1 (NL4-3 based) proviral constructs carrying functional *nef* genes followed by an internal ribosome entry site (IRES) and the eGFP gene has been described [25],[28]. Derivatives containing stop codons at positions 73 and 74 of the *nef* ORF either alone (nef*) or in combination with mutations in the ATG initiation codon and two in frame stop codons at positions four and five of the *nef* ORF (*nef*-) disrupting the NL4-3 nef gene were generated by standard PCR and cloning techniques. Splice-overlap-extension PCR was used to replace the NL4-3 *nef* gene with the bulks of SIVsmm *nef* genes. Briefly, PCR fragments containing the 3′end of the NL4-3 *env* gene fused to the various pools of SIVsmm *nef* genes were cloned into pBR-NL43-IRES-eGFP-*nef* ^+^ using the unique *HpaI* and *MluI* sites (Figure S1). Aliquots of transformed *Escherichia coli* were plated on Luria broth-ampicillin dishes to assess transformation efficiency, and the remaining 90% of the transformed bacteria were used for direct inoculation of medium-scale plasmid preparations. The percentage of the plasmid population containing an SIVsmm *nef* insert was estimated by restriction analysis and the integrity of all PCR-derived inserts was confirmed by sequence analysis. Nef alleles from 19 of the 26 SMs have been previously analyzed in transient transfection experiments [28]. Synthetic *nef* alleles, i.e. SMhi, SMlow, FCs clone 1mut and FBr clone 17mut, fused to the 3′end of the HIV-1 *env* gene were synthesized by Epoch Biolabs and cloned into pBR-NL43-IRES-eGFP vector using the unique *HpaI* and *MluI* sites.

### Cell culture

Jurkat and 293T-cells were cultured as described previously [28]. 293T-cells were maintained in Dulbecco's modified Eagle's medium containing 10% heat-inactivated fetal bovine serum. PBMC from healthy human donors were isolated using lymphocyte separation medium (Biocoll Separating Solution, Biochrom), stimulated for 3 days with PHA (2 µg/ml) and cultured in RPMI1640 medium with 10% FCS and 10 ng/ml IL-2 prior to infection. CD8^+^ T cells were depleted from the PBMC cultures using CD8 MicroBeads (Miltenyi Biotec) following the MACS separation protocol provided by the manufacturer.

### Virus stocks and transductions

To generate viral stocks, 293T cells were co-transfected with the proviral HIV-1 constructs and a plasmid (pHIT-G) expressing the Vesicular Stomatitis Virus G protein (VSG-G) [25],[28]. The latter was used to achieve comparable high initial infection levels for functional analysis. However, all HIV-1 constructs contained intact *env* genes and were thus replication competent following the first round of infection. The medium was changed after overnight incubation and virus was harvested 24 h later. Residual cells in the supernatants were pelleted and the supernatants were stored at −70°C. Virus stocks were quantified using a p24 antigen capture assay provided by the NIH AIDS Research and Reference Reagent Program.

### Infectivity assays

Virus infectivity was determined using TZM-bl and P4-CCR5 cells as described [65]. Briefly, the cells were sown out in 96-well-dishes in a volume of 100 µl and infected after overnight incubation with virus stocks containing 1 of p24 antigen produced by transiently transfected 293T cells. Two days post-infection viral infectivity was detected using the Gal screen kit from TROPIX as recommended by the manufacturer. β-galactosidase activities were quantified as relative light units per second (RLU/s) using the Orion Microplate Luminomter.

### Flow cytometric analysis

CD4, TCR-CD3, MHC-I, CD28 and eGFP reporter expression in Jurkat T cells or human PBMC transduced with HIV-1 (NL4-3) constructs coexpressing Nef and eGFP were measured as described [25],[28]. CD8, IL-2R, CD69 and PD-1 expression was measured by standard FACS staining, using CD8 (BD Pharmingen, Clone RPA-T8), CD25 (BD Pharmingen, Clone M-A251), CD69 (BD Pharmingen, Clone FN50) and PD-1 (BD Pharmingen, Clone MIH4) mAbs. For quantification of Nef-mediated modulation of specific surface molecules, the levels of receptor expression (red fluorescence) were determined for cells expressing a specific range of eGFP. The extent of downmodulation (*n*-fold) was calculated by dividing the MFI obtained for cells infected with the *nef*-minus NL4-3 control viruses by the corresponding values obtained for cells infected with viruses coexpressing Nef and eGFP.

### PBMC activation and apoptosis

Human PBMC were first stimulated with PHA (1 µg/ml) for 3 days. Subsequently, the cells were cultured in RPMI1640 (10% FCS, 10 ng/ml IL-2), infected with various HIV-1 eGFP/Nef constructs and cultured for another 2 days. At this time the PBMC expressed very low levels of CD69 and IL-2R and hence had a resting phenotype. Thereafter, the PBMC were treated a second time with PHA and CD69 and IL-2R expression levels were measured by FACS analysis one and four days later. The frequency of virally infected apoptotic cells was determined using the AnnexinV (AnV) Apoptosis Detection Kit (BD Bioscience) as recommended by the manufacturer.

### NFAT induction

Jurkat cells stably transfected with an NFAT-dependent reporter gene vector [31] were either left uninfected or transduced with HIV-1 Nef/eGFP constructs expressing various *nef* alleles. Except for those cells used as controls, cultures were treated with PHA (1 µg/ml; Murex). Luciferase activity was measured and *n*-fold induction determined by calculating the ratio between measured relative light units of treated samples over untreated samples as described previously [28],[31].

### Statistical analysis

The activities of *nef* alleles derived from SMs with low (n = 15) or high (n = 11) CD4^+^ T cell counts and of the subgroups of animals expressing *nef* alleles showing the highest (n = 6) or lowest (n = 6) activities in CD3 and MHC-I modulation were compared using a two-tailed Student's t test. The PRISM package version 4.0 (Abacus Concepts, Berkeley, CA) was used for all calculations.

### Accession numbers

GenBank accession numbers for previously described SIVsmm *nef* sequences are DQ408682 to DQ408725 and for newly derived sequences EU636907 to EU636923 (see Tables S1 and S2 for further detail).

## Supporting Information

Figure S1 Generation of HIV-1 NL4-3 constructs expressing heterologous SIVsmm *nef* alleles. SIVsmm *nef* alleles were directly amplified from plasma of infected SMs by RT-PCR [27]. For cloning into the proviral NL4-3 IRES/eGFP constructs, the 3′ end of the HIV-1 *env* gene and the SIVsmm *nef* genes were fused by splice-overlap-extension (SOE) PCR using outer primers containing *HpaI* and *MluI* restriction sites and overlapping inner primers and cloned in bulk into the proviral constructs. The resulting proviral constructs differed specifically in their *nef* genes.
(0.10 MB TIF)

Figure S2 SIVsmm Nef-mediated receptor modulation in Jurkat cells and CD8-depleted PBMCs. Surface expression of CD3, CD4 and MHC-I on Jurkat cells (upper) and CD8-depleted PBMCs (lower) infected with HIV-1 Nef/eGFP constructs expressing eGFP alone (*nef*-) or together with indicated *nef* alleles. 239 specifies the SIVmac239 Nef. Similar results were obtained in two independent experiments.
(0.75 MB TIF)

Figure S3 Correlation between the efficiencies of Nef-mediated CD4, CD3 and MHC-I downmodulation in Jurkat cells, PBMCs and CD8-depleted PBMCs.
(0.10 MB TIF)

Figure S4 Correlation between the efficiency of CD3 and MHC-I downmodulation by Nef and the number of CD4^+^ T cells in SIVsmm infected SMs. Correlation between (A, C) the absolute numbers and (B, D) the percentages of CD4^+^ T-cells in SIVsmm-infected SMs and *n*-fold downmodulation of CD3 and MHC-I or the sum thereof. Nef function was determined in PBMCs in both the presence and absence of CD8^+^ T cells. Nef alleles from the CD4high and CD4low groups of SMs are color coded green and red, respectively.
(0.19 MB TIF)

Figure S5 Correlation between the efficiency of CD3 and MHC-I downmodulation by Nef in CD8-depleted PBMCs and immological features in SIVsmm infected SMs. Correlation between (A) the number of proliferating CD4^+^Ki67^+^ T cells, (B) CD95 expression levels of CD4^+^ T-cells, (C) number of CD8^+^CD28^+^ T cells and (C) percentage of naïve CD4^+^ T cells in SIVsmm-infected SMs and *n*-fold downmodulation of CD3 and MHC-I. Assays were performed in PBMCs depleted of CD8^+^ T cells. Data derived from *nef* alleles derived from the CD4high and CD4low groups of SMs are color coded green and red, respectively and were confirmed in two independent experiments.
(0.15 MB TIF)

Figure S6 Effect of SIVsmm Nef alleles on the activation of CD8-depleted PBMCs. (A) Levels of CD69, PD-1 and IL-2R expression on CD8-depleted PBMCs transduced with HIV-1 constructs expressing Nef alleles from SIVsmm-infected SMs with low (red) or high (green) CD4^+^ T-cell counts. The mean fluoresence activities (MFIs) of CD69, PD-1 and IL-2R expression were determined at 1 and 2 days post-stimulation, respectively. Correlation between the efficiency of Nef-mediated CD3 downmodulation and (B) IL-2R, (C) PD-1 and (D) IL-2R surface expression levels. Correlation between (E) PD-1 and CD69 and (F) PD-1 and IL-2R expression. The results were confirmed in two independent experiments.
(0.20 MB TIF)

Figure S7 Comparison of SIVsmm Nef alleles differing in their ability to modulate cellular receptors and the responsiveness of T cells to activation. Alignment of amino acid sequences of (A) the SMhi and SMlow nef alleles, (B) the primary FCs clone 1 and FYb clone 17 *nef* genes and two mutants thereof and (C) primary FBr *nef* alleles. Mutagenized residues are indicated in bold and dots specify amino acid identity.
(1.41 MB TIF)

Table S1 Functional activity of primary SIVsmm Nef alleles tested in bulk. Functional activity of primary SIVsmm Nef alleles tested in bulk. Nef-mediated *n*-fold receptor downmodulation was calculated by dividing the MFI of cells infected with a *nef*-deficient HIV-1 expressing a specific range of green fluorescence by the corresponding number obtained for cells infected with HIV-1 constructs coexpressing Nef and eGFP. The results were confirmed in three to five independent experiments. Viral infectivity is given relative to that of SIVmac239 (100%) and represents averages ±SD (n = 6). The levels of NFAT-dependent luciferase reporter activity in Jurkat cells infected with *nef*-defective HIV-1 were set to 100%. Shown are averages (±SD) of triple infections.
(0.01 MB PDF)

Table S2 Functional activity of primary and synthetic mutant SIVsmm nef alleles. Functional activity of primary and synthetic mutant SIVsmm *nef* alleles. Receptor modulation was calculated as described in the legend to Table S1. The levels of CD69, PD-1 and IL-2R surface expression, of NFAT-dependent luciferase reporter activities and of apoptotic PBMCs measured in cell cultures infected with the *nef*-defective control virus were set to 100%. Abbreviations: G1, G2, Group 1 Nefs do not, and Group 2 Nefs do downregulate TCR-CD3. All values are averages derived from two to four experiments.
(0.01 MB PDF)
