# Supplementary figures and images for: Inefficient Nef-Mediated Downmodulation of CD3 and MHC-I Correlates with Loss of CD4+ T Cells in Natural SIV Infection
Source: PLoS Pathog. 2008 Jul 18;4(7):e1000107. doi: 10.1371/journal.ppat.1000107 (PMC2444047; doi:10.1371/journal.ppat.1000107)

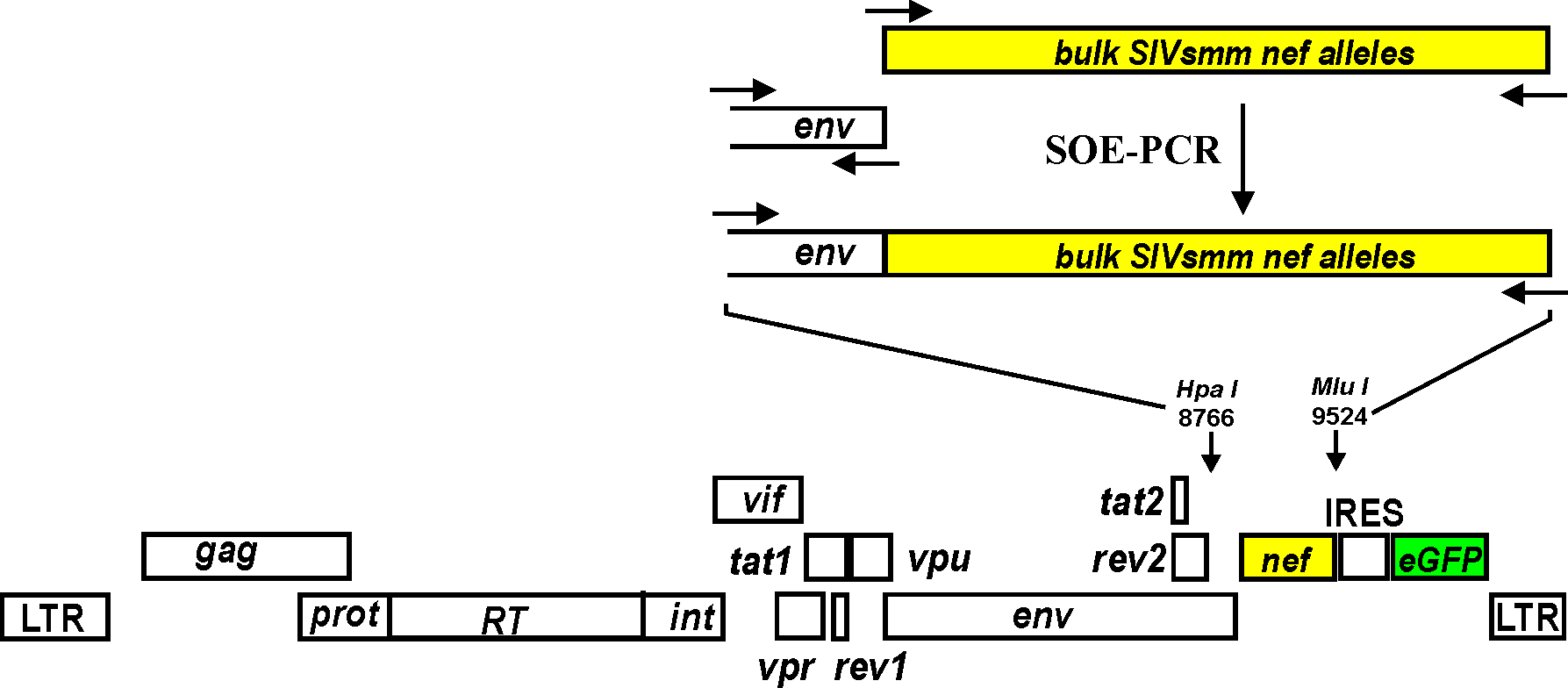

Supplement: Figure S1 — Generation of HIV-1 NL4-3 constructs expressing heterologous SIVsmm nef alleles. SIVsmm nef alleles were directly amplified from plasma of infected SMs by RT-PCR [27]. For cloning into the proviral NL4-3 IRES/eGFP constructs, the 3′ end of the HIV-1 env gene and the SIVsmm nef genes were fused by splice-overlap-extension (SOE) PCR using outer primers containing HpaI and MluI restriction sites and overlapping inner primers and cloned in bulk into the proviral constructs. The resulting proviral constructs differed specifically in their nef genes. (0.10 MB TIF) [file ppat.1000107.s001.tif]

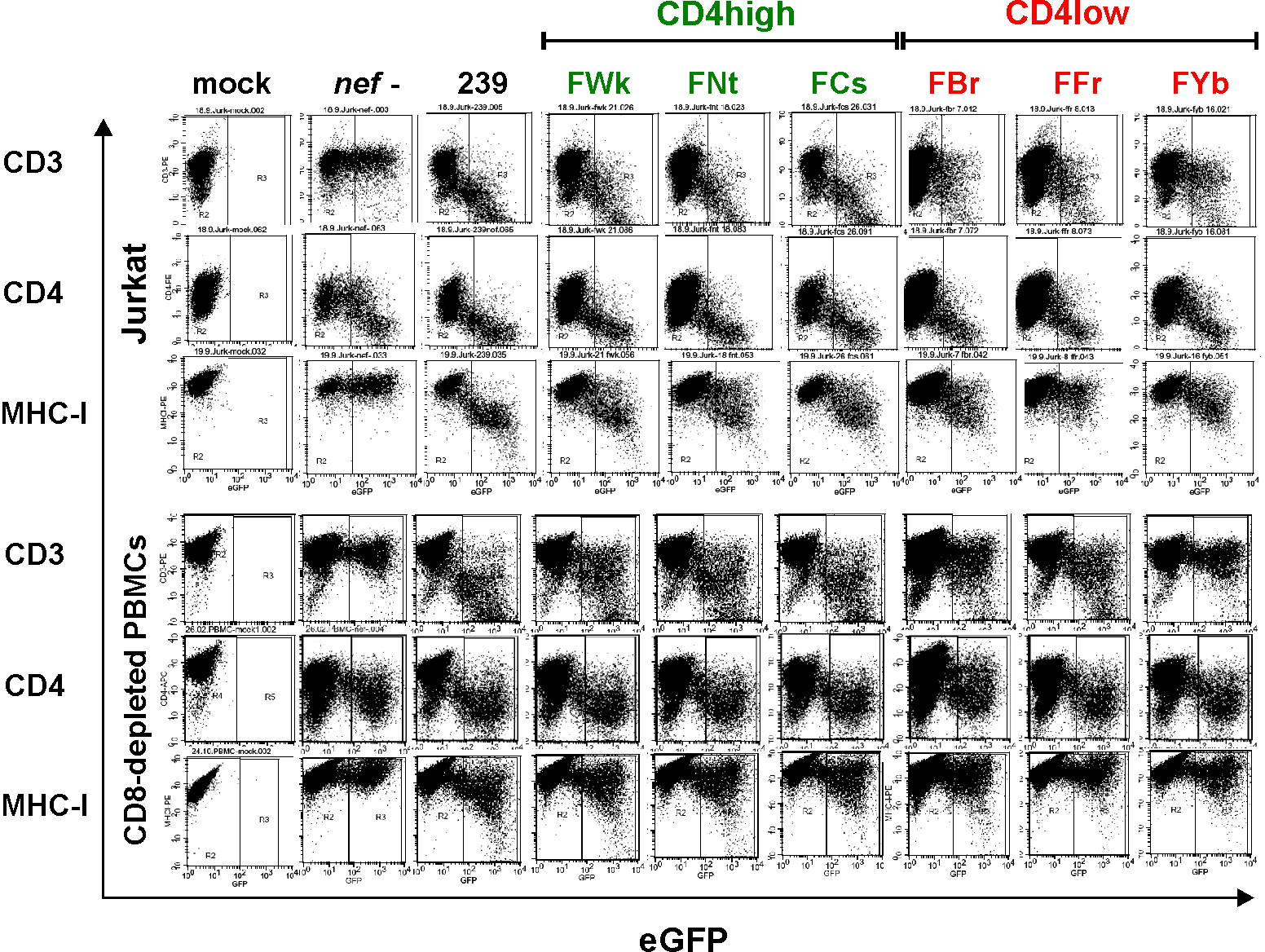

Supplement: Figure S2 — SIVsmm Nef-mediated receptor modulation in Jurkat cells and CD8-depleted PBMCs. Surface expression of CD3, CD4 and MHC-I on Jurkat cells (upper) and CD8-depleted PBMCs (lower) infected with HIV-1 Nef/eGFP constructs expressing eGFP alone (nef-) or together with indicated nef alleles. 239 specifies the SIVmac239 Nef. Similar results were obtained in two independent experiments. (0.75 MB TIF) [file ppat.1000107.s002.tif]

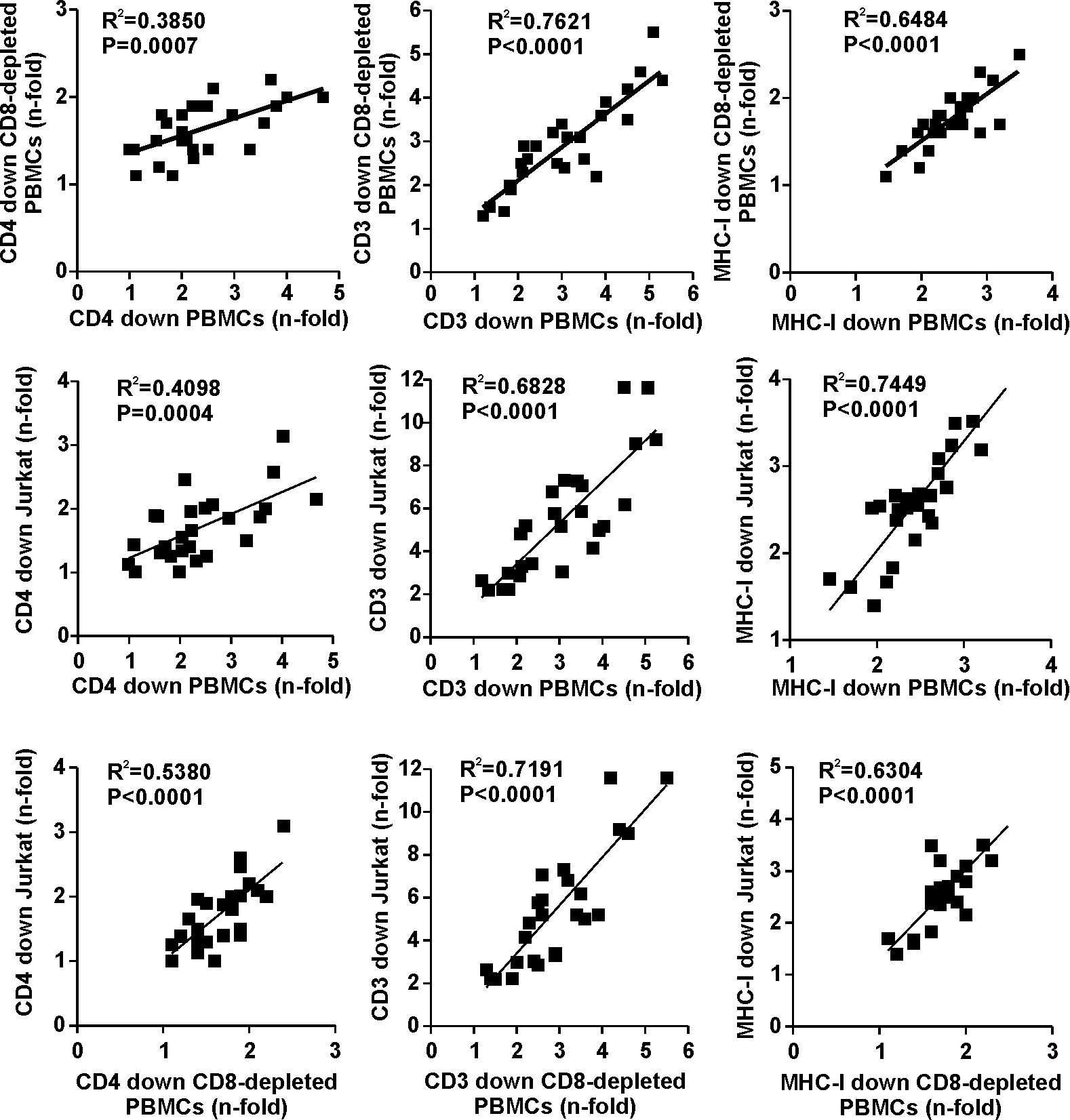

Supplement: Figure S3 — Correlation between the efficiencies of Nef-mediated CD4, CD3 and MHC-I downmodulation in Jurkat cells, PBMCs and CD8-depleted PBMCs. (0.10 MB TIF) [file ppat.1000107.s003.tif]

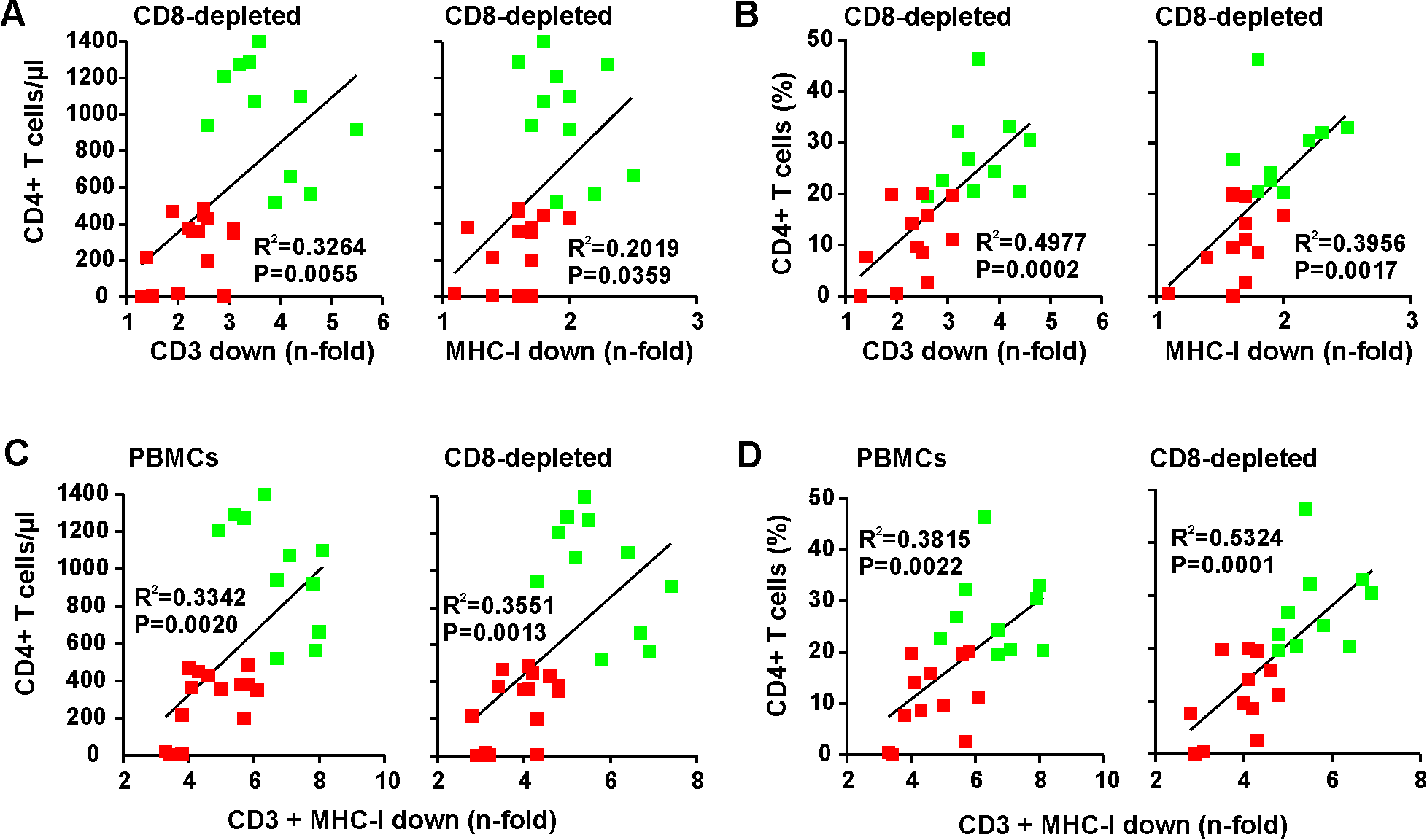

Supplement: Figure S4 — Correlation between the efficiency of CD3 and MHC-I downmodulation by Nef and the number of CD4+ T cells in SIVsmm infected SMs. Correlation between (A, C) the absolute numbers and (B, D) the percentages of CD4+ T-cells in SIVsmm-infected SMs and n-fold downmodulation of CD3 and MHC-I or the sum thereof. Nef function was determined in PBMCs in both the presence and absence of CD8+ T cells. Nef alleles from the CD4high and CD4low groups of SMs are color coded green and red, respectively. (0.19 MB TIF) [file ppat.1000107.s004.tif]

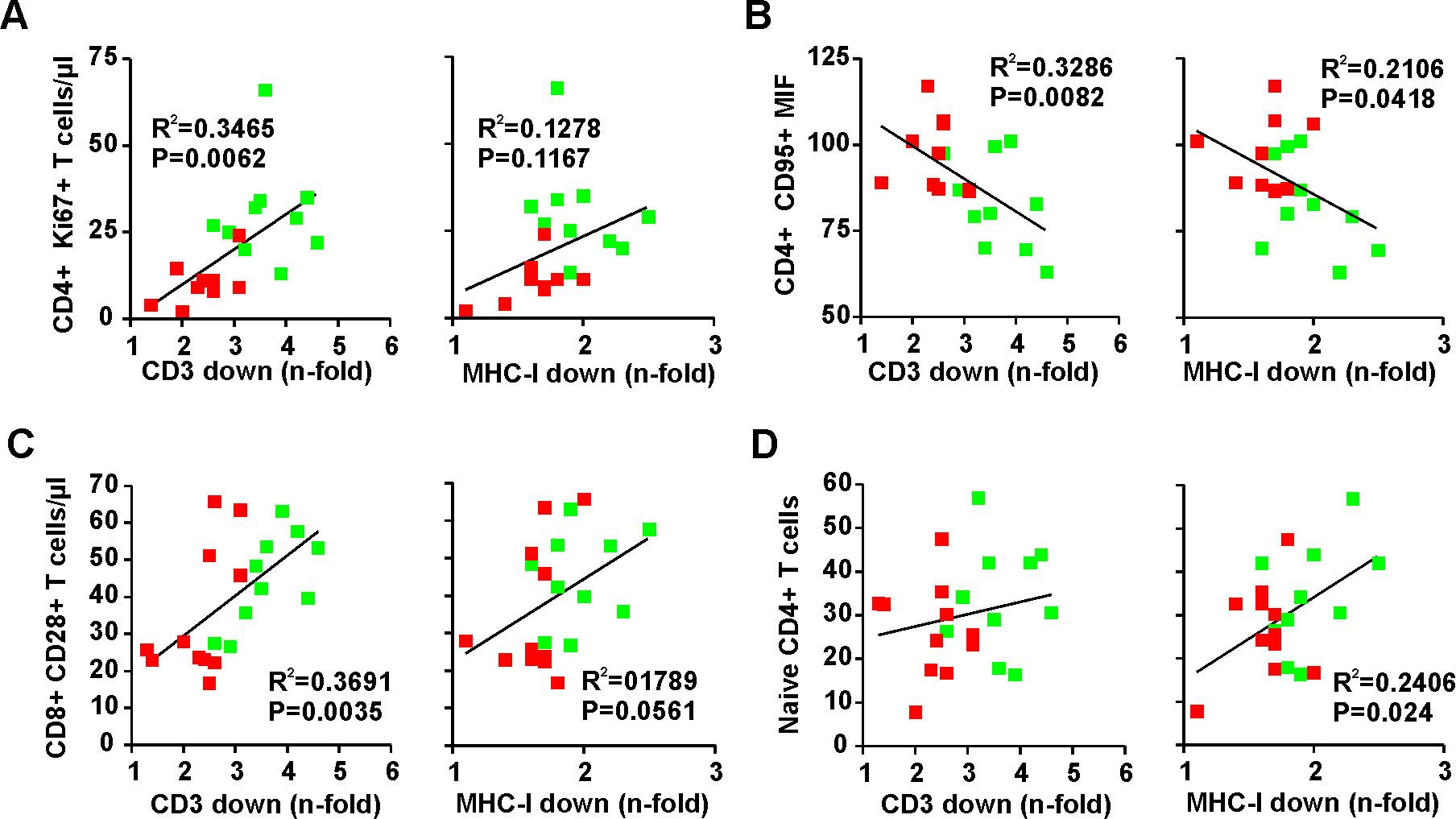

Supplement: Figure S5 — Correlation between the efficiency of CD3 and MHC-I downmodulation by Nef in CD8-depleted PBMCs and immological features in SIVsmm infected SMs. Correlation between (A) the number of proliferating CD4+Ki67+ T cells, (B) CD95 expression levels of CD4+ T-cells, (C) number of CD8+CD28+ T cells and (C) percentage of naïve CD4+ T cells in SIVsmm-infected SMs and n-fold downmodulation of CD3 and MHC-I. Assays were performed in PBMCs depleted of CD8+ T cells. Data derived from nef alleles derived from the CD4high and CD4low groups of SMs are color coded green and red, respectively and were confirmed in two independent experiments. (0.15 MB TIF) [file ppat.1000107.s005.tif]

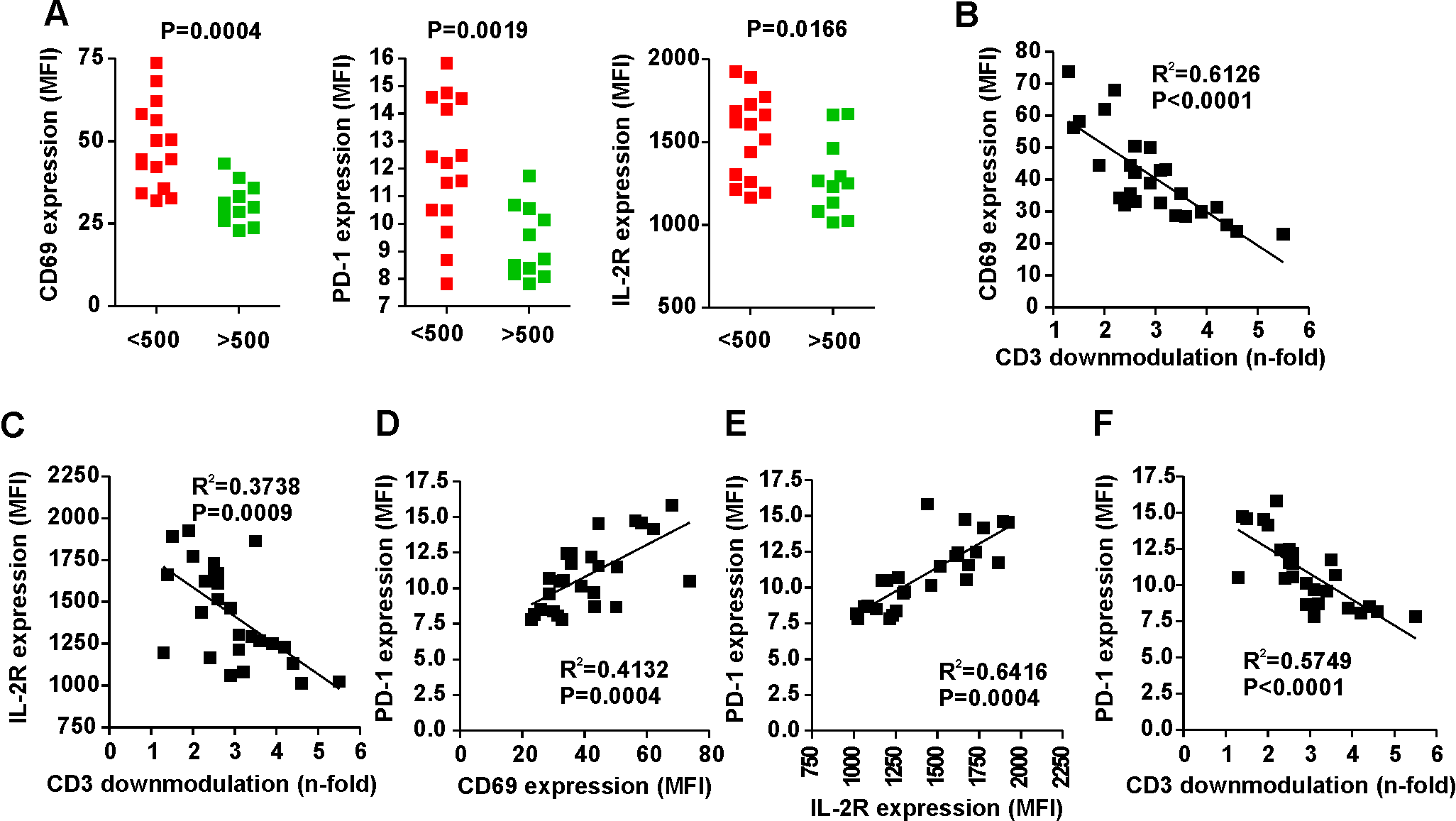

Supplement: Figure S6 — Effect of SIVsmm Nef alleles on the activation of CD8-depleted PBMCs. (A) Levels of CD69, PD-1 and IL-2R expression on CD8-depleted PBMCs transduced with HIV-1 constructs expressing Nef alleles from SIVsmm-infected SMs with low (red) or high (green) CD4+ T-cell counts. The mean fluoresence activities (MFIs) of CD69, PD-1 and IL-2R expression were determined at 1 and 2 days post-stimulation, respectively. Correlation between the efficiency of Nef-mediated CD3 downmodulation and (B) IL-2R, (C) PD-1 and (D) IL-2R surface expression levels. Correlation between (E) PD-1 and CD69 and (F) PD-1 and IL-2R expression. The results were confirmed in two independent experiments. (0.20 MB TIF) [file ppat.1000107.s006.tif]

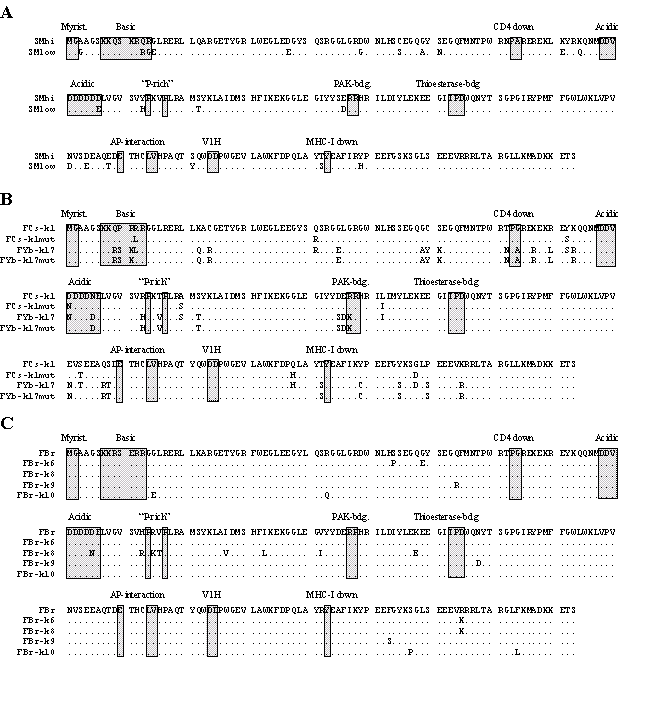

Supplement: Figure S7 — Comparison of SIVsmm Nef alleles differing in their ability to modulate cellular receptors and the responsiveness of T cells to activation. Alignment of amino acid sequences of (A) the SMhi and SMlow nef alleles, (B) the primary FCs clone 1 and FYb clone 17 nef genes and two mutants thereof and (C) primary FBr nef alleles. Mutagenized residues are indicated in bold and dots specify amino acid identity. (1.41 MB TIF) [file ppat.1000107.s007.tif]
