# Supplementary material for: Inefficient Nef-Mediated Downmodulation of CD3 and MHC-I Correlates with Loss of CD4+ T Cells in Natural SIV Infection
Source: PLoS Pathog. 2008 Jul 18;4(7):e1000107. doi: 10.1371/journal.ppat.1000107 (PMC2444047; doi:10.1371/journal.ppat.1000107)
Supplement: Table S1 — Functional activity of primary SIVsmm Nef alleles tested in bulk. Functional activity of primary SIVsmm Nef alleles tested in bulk. Nef-mediated n-fold receptor downmodulation was calculated by dividing the MFI of cells infected with a nef-deficient HIV-1 expressing a specific range of green fluorescence by the corresponding number obtained for cells infected with HIV-1 constructs coexpressing Nef and eGFP. The results were confirmed in three to five independent experiments. Viral infectivity is given relative to that of SIVmac239 (100%) and represents averages ±SD (n = 6). The levels of NFAT-dependent luciferase reporter activity in Jurkat cells infected with nef-defective HIV-1 were set to 100%. Shown are averages (±SD) of triple infections. (0.01 MB PDF) [file ppat.1000107.s008.pdf]

Table S1. Functional activity of primary SIVsmm Nef alleles tested in bulk

SM	CD4+ T cell count/µl	GenBank Accession numbers	down-modulation (n-fold) of: CD4	CD3	CD28	MHC-I	CXCR4	Infectivity (%) P4-CCR5	TZM-bl	NFAT-dep. Luc activity (%)
FNp	1400	DQ408682, DQ408683	2,3	3,9	1,8	2,3	1,5	45,3±4,0	51,3±4,6	36,3±3,3
FZs	1291	DQ408716, DQ408717	1,7	3,0	1,4	2,3	1,0	74,8±14,3	90,5±9,8	18,2±2,8
FWk	1275	DQ408718, DQ408719	3,7	2,8	2,2	2,9	1,9	71,4±12,8	111,7±3,8	60,0±3,2
FTn	1210	DQ408714, DQ408715	2,0	2,4	1,5	2,6	1,9	61,6±18,3	86,7±1,6	20,0±4,1
FNt	1100	DQ408712, DQ408713	4,7	5,3	1,8	2,8	1,6	91,6±10,7	132,0±9,9	27,0±3,7
FJj	1073	EU636911, EU636912	2,0	4,5	1,9	2,6	1,2	15,6±4,0	8,1±0,8	31,8±9,0
FHr	941	DQ408710, DQ408711	2,5	3,5	1,6	3,2	1,2	57,8±4,9	86,2±3,8	26,7±8,5
FCs	917	EU636913, EU636914	2,6	5,1	1,8	2,7	1,3	108,3±20,7	110,4±24,9	33,3±0,3
FJn	663	DQ408702, DQ408703	4,0	4,5	2,1	3,5	1,4	104,3±17,5	121,4±9,6	28,3±0,3
FAi	564	DQ408720, DQ408721	3,8	4,8	1,9	3,1	1,9	222,8±24,3	191,1±47,9	48,8±4,1
FKl	520	DQ408722, DQ408723	2,0	4,0	1,8	2,7	1,6	36,7±4,0	74,9±16,7	36,8±4,2
FEm	486	DQ408688, DQ408689	2,1	2,9	2,4	2,9	1,3	62,6±7,6	57,1±4,3	33,3±3,5
FFv	468	DQ408698, DQ408699	1,1	1,8	1,3	2,2	0,6	10,5±0,6	18,1±4,2	40,7±7,1
FIg	450	DQ408700, DQ408701	1,0	2,1	1,9	2,3	1,7	83,8±8,3	82,4±7,0	59,0±11,8
FBn	431	DQ408696, DQ408697	2,2	2,2	1,4	2,4	1,2	31,7±5,5	31,2±8,7	54,2±6,6
FJo	380	DQ408704, DQ408705	2,5	3,1	1,7	2,5	1,6	68,4±11,1	105,3±14,4	56,1±5,2
FCq	379	DQ408724, DQ408725	3,3	3,8	1,6	2,0	1,2	29,6±0,5	32,0±1,0	69,2±3,8
FNg	363	DQ408686, DQ408687	1,5	2,1	1,6	2,0	0,7	29,6±2,7	71,3±12,9	50,8±5,7
FDn	357	DQ408684, DQ408685	2,2	3,1	1,5	1,9	1,0	37,2±5,8	62,1±7,7	47,3±3,5
FPm	351	DQ408706, DQ408707	3,6	3,4	2,1	2,6	1,4	75,0±0,2	145,5±42,4	53,2±5,6
FFj	218	DQ408690, DQ408691	1,8	1,7	1,3	2,1	1,1	36,3±5,7	75,8±6,0	37,0±12,1
FAn	200	EU636909, EU636910	2,2	3,5	2,4	2,2	1,1	58,8±10,2	90,0±4,1	45,2±11,3
FRg	20	EU636907, EU636908	1,1	1,8	1,0	1,5	1,0	37,6±11,7	66,8±7,5	32,7±7,3
FFr	7	DQ408694, DQ408695	1,6	2,1	1,5	1,7	1,3	93,5±11,7	120,7±5,1	48,5±8,0
FBr	5	DQ408692, DQ408693	1,6	1,4	1,9	2,5	2,1	6,9±1,6	24,6±5,5	48,5±13,6
FYb	3	DQ408708, DQ408709	3,0	1,2	1,1	2,2	0,8	55,1±16,9	67,7±15,7	239,2±13,9
