# Supplementary material for: Inefficient Nef-Mediated Downmodulation of CD3 and MHC-I Correlates with Loss of CD4+ T Cells in Natural SIV Infection
Source: PLoS Pathog. 2008 Jul 18;4(7):e1000107. doi: 10.1371/journal.ppat.1000107 (PMC2444047; doi:10.1371/journal.ppat.1000107)
Supplement: Table S2 — Functional activity of primary and synthetic mutant SIVsmm nef alleles. Functional activity of primary and synthetic mutant SIVsmm nef alleles. Receptor modulation was calculated as described in the legend to Table S1. The levels of CD69, PD-1 and IL-2R surface expression, of NFAT-dependent luciferase reporter activities and of apoptotic PBMCs measured in cell cultures infected with the nef-defective control virus were set to 100%. Abbreviations: G1, G2, Group 1 Nefs do not, and Group 2 Nefs do downregulate TCR-CD3. All values are averages derived from two to four experiments. (0.01 MB PDF) [file ppat.1000107.s009.pdf]

Table S2. Functional activity of primary and synthetic mutant SIVsmm *nef* alleles

Allele	GenBank Accession #	Remark	downmodulation (n-fold) CD4	CD3	CD28	MHC-I	expression (%) CD69	IL-2R	Apotosis (%)	NFAT-dep. Luc activity (%)
NL4-3	M19921	Control G1	2,6	1,0	1,8	2,5	94,4	77,8	151,3	397,8±97,9
mac239	M33262	Control G2	2,6	5,7	5,7	3,3	10,1	52,4	57,2	23,2±4,7
SMhi	EU636915	Synthetic allele	2,5	6,1	4,8	3,9	8,6	43,3	53,4	33,0±4,7
SMlow	EU636916	Synthetic allele	2,5	4,5	4,1	3,1	14,1	60,3	64,2	71,8±3,7
FCs clone 1	EU636913	Primary allele	2,2	4,2	2,1	2,4	24,0	62,3	85,6	57,3±7,7
FCs cl.1mut	EU636917	Synthetic allele	1,4	1,0	1,3	1,2	92,3	90,0	112,9	737,6±30,8
FYb clone 17	EU636918	Primary allele	1,5	1,6	1,4	2,3	59,0	77,3	157,0	560,8±60,7
FYb cl.17mut	EU636919	Synthetic allele	1,3	2,9	2,1	2,4	20,2	63,1	65,4	64,0±9,6
FBr clone 6	EU636920	Primary allele	2,3	5,8	11,9	4,0	15,5	36,1	53,5	62,1±6,3
FBr clone 8	EU636921	Primary allele	2,5	1,1	3,1	2,6	112,1	80,1	85,0	175,7±15,6
FBr clone 9	EU636922	Primary allele	2,1	5,9	1,9	1,6	36,7	57,4	n.d.	44,7±1,6
FBr clone 10	EU636923	Primary allele	2,7	5,6	7,9	1,6	49,4	47,3	n.d	44,8±3,3
